# Geodesic Convexity of the Symmetric Eigenvalue Problem and Convergence of Steepest Descent

**DOI:** 10.1007/s10957-024-02538-8

**Published:** 2024-10-08

**Authors:** Foivos Alimisis, Bart Vandereycken

**Affiliations:** Department of Mathematics, University of Geneva, Geneva, Switzerland

**Keywords:** Block Rayleigh quotient, Grassmann manifold, Geodesic convexity, Riemannian optimization, Low-rank approximation

## Abstract

We study the convergence of the Riemannian steepest descent algorithm on the Grassmann manifold for minimizing the block version of the Rayleigh quotient of a symmetric matrix. Even though this problem is non-convex in the Euclidean sense and only very locally convex in the Riemannian sense, we discover a structure for this problem that is similar to geodesic strong convexity, namely, weak-strong convexity. This allows us to apply similar arguments from convex optimization when studying the convergence of the steepest descent algorithm but with initialization conditions that do not depend on the eigengap $\delta$. When $\delta >0$, we prove exponential convergence rates, while otherwise the convergence is algebraic. Additionally, we prove that this problem is geodesically convex in a neighbourhood of the global minimizer of radius ${\mathcal {O}}(\sqrt{\delta })$.

## Introduction

We consider the problem of computing the top *k* eigenvectors of a symmetric matrix $A \in \mathbb {R}^{n \times n}$, which has many applications in numerical linear algebra (low rank approximation), statistics (principal component analysis) and signal processing. Without loss of generality, we assume that *A* is also positive semidefinite. This is because *A* can be shifted as $A+c I_n$ for some constant *c* and this transformation does not change its eigenvectors.

We denote by $\lambda _1 \ge \lambda _2 \ge \cdots \ge \lambda _n$ the eigenvalues of *A* counted with multiplicity and by $\delta :=\lambda _k-\lambda _{k+1}$ the eigengap for some *k* between 1 and $n-1$. We also denote $\Lambda _{\alpha } = \text {diag}(\lambda _1, \ldots ,\lambda _k)$ and $\Lambda _{\beta } = \text {diag}(\lambda _{k+1}, \ldots ,\lambda _n)$.

A set of *k* leading eigenvectors of *A* can be found by minimizing the function

$$\begin{aligned} f(X) = -{{\,\textrm{Tr}\,}}(X^TAX) \end{aligned}$$

over the set of $n \times k$ matrices with orthonormal columns. Indeed, from Fan’s trace minimization theorem (see, e.g., [18, Corollary 4.3.39]) we know that

1 $$\begin{aligned} \min \{ f(X) :X \in \mathbb {R}^{n \times k}, X^T X = I_k \} = -(\lambda _1 + \cdots + \lambda _k)=-{{\,\textrm{Tr}\,}}(\Lambda _{\alpha })=:f^*. \end{aligned}$$

Since *A* is symmetric, we can define the matrix $$V_{\alpha } = \begin{bmatrix} v_1 \hspace{2mm} \cdots \hspace{2mm} v_k \end{bmatrix}$$ such that $V_{\alpha }^T V_{\alpha } = I_k$ and with $v_i \in \mathbb {R}^n$ a unit-norm eigenvector corresponding to $\lambda _i$. If the eigengap $\delta$ is strictly positive, then ${{\,\textrm{span}\,}}(V_{\alpha })$ is unique; otherwise, we can choose any $v_k$ from a subspace with dimension equal to the multiplicity of $\lambda _k$. It is readily seen that $f(V_{\alpha })= -(\lambda _1 + \cdots + \lambda _k)$. In fact, all minimizers of (1) are of the form $V_{\alpha } Q$ with *Q* a $k \times k$ orthogonal matrix. We also define $$V_{\beta }=\begin{bmatrix} v_{k+1} \hspace{2mm} \cdots \hspace{2mm} v_n \end{bmatrix}$$ that contains the eigenvectors corresponding to the eigenvalues $\lambda _{k+1},\ldots ,\lambda _n$. Its columns span the orthogonal complement of ${{\,\textrm{span}\,}}(V_{\alpha })$ in $\mathbb {R}^n$ and thus $V_{\beta }^T V_{\beta } = I_{n-k}$ and $V_{\alpha }^T V_{\beta } = {0}_{k \times (n-k)}$.

Since ${{\,\textrm{span}\,}}(V_{\alpha }) = {{\,\textrm{span}\,}}(V_{\alpha }Q)$, it is more natural to consider this problem as a minimization problem on the Grassmann manifold ${{\,\textrm{Gr}\,}}(n,k)$, the set of *k*-dimensional subspaces in $\mathbb {R}^n$. Let us therefore redefine the objective function as

2 $$\begin{aligned} f(\mathcal {X}) = -{{\,\textrm{Tr}\,}}(X^TAX) \text { where}\,\mathcal {X} = {{\,\textrm{span}\,}}(X) \hbox { for }X \in \mathbb {R}^{n \times k}\hbox { s.t. }X^T X = I_k. \end{aligned}$$

This cost function can be seen as a block version of the standard Rayleigh quotient. An immediate benefit is that, if $\delta > 0$, the minimizer of (2) is isolated since it is the subspace $\mathcal {V_{\alpha }} = {{\,\textrm{span}\,}}(V_\alpha )$.

To minimize *f* on ${{\,\textrm{Gr}\,}}(n,k)$, we shall use the Riemannian steepest descent method (RSD) along geodesics in ${{\,\textrm{Gr}\,}}(n,k)$. Quite remarkably, for ${{\,\textrm{Gr}\,}}(n,k)$ these geodesics can be implemented efficiently in closed form.

For analyzing the convergence properties of steepest descent on ${{\,\textrm{Gr}\,}}(n,k)$, we extend results of the recent work [4], where it is shown that the Rayleigh quotient on the sphere enjoys favourable geodesic convexity-like properties, namely, *weak-quasi-convexity* and *quadratic growth*. In this work, we show that these convexity-like properties continue to hold in the more general case of the block Rayleigh quotient function $f:{{\,\textrm{Gr}\,}}(n,k) \rightarrow \mathbb {R}$. These results are of general interest, but also sufficient to prove a local convergence rate for steepest descent for minimizing *f* when started from an initial point outside the region of local convexity. For the latter, a crucial help is provided by the fact that the Grassmann manifold is non-negatively curved (see [33]).

In particular, assuming a *strictly positive eigengap* $\delta$ between $\lambda _k$ and $\lambda _{k+1}$, we prove an exponential convergence rate to the subspace spanned by the *k* leading eigenvectors, similar to the convergence of power method and subspace iteration (Theorem 5.1). If we do not assume any knowledge regarding the eigengap, then we can still prove a sub-exponential (polynomial) convergence rate of the function values to the global minimum (Theorem 5.2), but we cannot directly study the convergence to a global minimizer. This is in line with previous work but our analysis does not use standard notions of geodesic convexity and allows for an initial guess further from the global minimizer. In Appendix B we present related convergence results for steepest descent with a more tractable step size but at the expense of needing a slightly better initialization.

## Related Work

The symmetric eigenvalue problem has been popular for several decades in the numerical linear algebra and optimization communities. When only a few eigenvalues are targeted, the main solvers for this problem have been based on subspace iteration and Krylov subspace methods. Less but still considerable attention has been given to the steepest descent method and its accelerated versions. Most works on steepest descent focus only on computing the first leading eigenvector of a symmetric matrix ($k=1$), using a Euclidean version of the algorithm. Asymptotic convergence rates are known for this setting since the 1950’s, see [15]. More recently, exact non-asymptotic estimates for the same Euclidean steepest descent with exact line search were proved in [20]. For a more comprehensive overview of this line of research, the reader can refer to [26] and the references therein. A recent result that takes a different route compared to the previous ones is [4]. There, a steepest descent algorithm on the sphere is analyzed using newly proved convexity-like properties of the spherical Rayleigh quotient.

Regarding the block version of the algorithm, where one targets multiple pairs of eigenvalues and eigenvectors, much less is known. We refer here to [27], which presents a steepest descent-like method for the multiple eigenvector problem using Ritz projections onto a 2*k*-dimensional subspace in each step. The convergence of this algorithm is proved to be linear, but computing the Ritz projections is quite expensive. Instead, in this work we consider a much cheaper version of steepest descent by directly choosing only one of the vectors in this 2*k*-dimensional subspace to update our algorithm. Some analysis for such a steepest descent (without Ritz projection) on the Grassmann manifold using a retraction and an Armijo step-size is provided in [2] (see Algorithm 3 and Theorem 4.9.1). Unfortunately this convergence rate is asymptotic, that is, a linear rate is achieved after an unknown number of iterations. The region in which the convergence happens cannot be quantified. Also, such a convergence rate does not yield an iteration complexity for the algorithm.

The optimization landscape provided by the block Rayleigh quotient on the Grassmann manifold has also received some attention lately. [32] provides many interesting properties of the critical points of this function and proves that all but the global optimum are strict saddles. This is later used to derive favourable convergence properties for a hybrid method consisting of Riemannian steepest descent in a first stage and a Riemannian Newton’s method in a final stage. [23] proves the so-called robust strict saddle property for this function, that is, the Hessian evaluated in each critical point except the global optimum has both positive and negative eigenvalues in a whole neighborhood. However, none of these papers talks about (generalized) convexity of any form, nor discusses any convergence rates for steepest descent.

Turning the discussion to the convexity properties of eigenvalue problems, there is a new line of research concerned by that. In [34], the authors prove (Theorem 4) that the Rayleigh quotient is geodesically gradient dominated in the sphere ($k=1$), that is, it satisfies a spherical version of the Polyak–Łojasiewicz inequality. In [4], it is shown that this result of [34] can be strengthened to a geodesic weak-quasi-convexity and quadratic growth property, which imply gradient dominance when combined. Finally, the recent paper [3] examines (among other contributions) the convexity structure of the same block version of the symmetric eigenvalue problem on the Grassmann manifold that we introduced above. Unfortunately, the characterization of the geodesic convexity region independently of the eigengap $\delta$ (Corollary 5 in [3]) is wrong (see our Appendix A for a counterexample). As we will prove in Theorem A.1, the geodesic convexity region of *f* (and the one of the equivalent cost function used in [3]) needs to depend on the eigengap, as appears also in [19, Lemma 7] in the case of the sphere ($k=1$).

To the best of our knowledge, the current work is the first that provides non-asymptotic convergence rates for the steepest descent algorithm for the multiple eigenvalue-eigenvector problem on the Grassmann manifold. We mainly rely on the work [4], which proves exponential convergence of steepest descent only in the case of $k=1$, that is, for the leading eigenvector. In this paper, we take a reasonable but highly non-trivial step forward by extending the convexity-like characterization of the spherical Rayleigh quotient to general *k*, that is, for a block of *k* leading eigenvectors. Again, the paper [9] is of high value for our current work regarding weakly-strongly-convex functions.

As mentioned above, the standard algorithm for computing the leading eigenspace of dimension *k* is subspace iteration (or power method when $k=1$).^1^ However, there are reasons to believe that, in certain cases, Riemannian steepest descent (and its accelerated version with non-linear conjugate gradients) should be preferred, especially in noisy settings [4] or in electronic structure calculations where the leading eigenspace of many varying matrices *A* needs to be computed.^2^ In particular, [4] presents strong experimental evidence that steepest descent is more robust to perturbations of the matrix–vector products than subspace iteration close to the optimum. While subspace iteration still behaves better at the start of the iteration, it asymptotically fails to converge to an approximation of the leading subspace that is as good as the one estimated by Riemannian steepest descent. While [4] dealt with a noisy situation due to calculations in a distributed setting with limited communication, exactly the same effect can be observed when we inject the matrix–vector products with Gaussian noise. Thus, we expect steepest descent to perform better than subspace iteration close to the optimum in any stochastic regime [14].

Regarding worst-case theoretical guarantees, the strongest convergence result for subspace iteration in the presence of a strictly positive eigengap $\delta$ is in terms of the largest principal angle between the iterates and the optimum [13], that is, the $\ell _\infty$-norm of the vector of principal angles. In contrast, our convergence result for steepest descent for $\delta > 0$ (Theorem 5.1) is in terms of the $\ell _2$-norm of the same vector of angles, which is in general stronger. When $\delta = 0$, it is known from [21, 28] that the largest eigenvalue ($k=1$) can still be efficiently estimated. We extend this result for $k>1$ and prove a convergence rate of steepest descent for the function values *f* (Theorem 5.2), relying only on weak-quasi-convexity (and thus using a different argument from [21, 28]).

## Geometry of the Grassmann Manifold and Block Rayleigh Quotient

We present here a brief introduction into the geometry of the Grassmann manifold. The content is not new and for more details, we refer to [2, 7, 12].

The (*n*, *k*)-Grassmann manifold is defined as the set of all *k*-dimensional subspaces of $\mathbb {R}^n$:

$$\begin{aligned} {{\,\textrm{Gr}\,}}(n,k)=\lbrace \mathcal {X} \subseteq \mathbb {R}^n :\mathcal {X} \hspace{1mm} \text {is a subspace and} \dim (\mathcal {X})=k \rbrace . \end{aligned}$$

Any element $\mathcal {X}$ of ${{\,\textrm{Gr}\,}}(n,k)$ can be represented by a matrix $X \in \mathbb {R}^{n \times k}$ that satisfies $\mathcal {X} = {{\,\textrm{span}\,}}(X)$. Such a representative is not unique since $Y=XQ$ for some invertible matrix $Q \in \mathbb {R}^{k \times k}$ satisfies ${{\,\textrm{span}\,}}(Y) = {{\,\textrm{span}\,}}(X)$. Without loss of generality, we will therefore always take matrix representatives *X* of subspaces $\mathcal {X}$ that have orthonormal columns throughout the paper. With some care, the non-uniqueness of the representatives is not a problem.^3^ For example, our objective function (2) is invariant to *Q*.

Riemannian structure. The set ${{\,\textrm{Gr}\,}}(n,k)$ admits the structure of a differential manifold with tangent spaces

3 $$\begin{aligned} T_{\mathcal {X}} {{\,\textrm{Gr}\,}}(n,k)=\lbrace G \in \mathbb {R}^{n \times k} :X^T G=0 \rbrace , \end{aligned}$$

where $\mathcal {X} = {{\,\textrm{span}\,}}(X)$. Since $X^T G = 0$ if and only if $(XQ)^T G=0$, for any invertible matrix $Q \in \mathbb {R}^{k \times k}$, this description of the tangent space does not depend on the representative *X*. However, a specific tangent vector *G* will depend on the chosen *X*. With slight abuse of notation,^4^ the above definition should therefore be interpreted as: given a fixed *X*, we define tangent vectors $G_1, G_2, \ldots$ of ${{\,\textrm{Gr}\,}}(n,k)$ at $\mathcal {X}={{\,\textrm{span}\,}}(X)$.

This subtlety is important, for example, when defining an inner product on $T_{\mathcal {X}} {{\,\textrm{Gr}\,}}(n,k)$:

$$\begin{aligned} \langle G_1, G_2 \rangle _{\mathcal {X}} = {{\,\textrm{Tr}\,}}(G^T_1 G_2) \ \text {\ with}\,G_1,G_2 \in T_{\mathcal {X}} {{\,\textrm{Gr}\,}}(n,k). \end{aligned}$$

Here, $G_1$ and $G_2$ are tangent vectors of the same representative *X*. Observe that the inner product is invariant to the choice of orthonormal representative: If $\bar{G}_1=G_1 Q$ and $\bar{G}_2 = G_2 Q$ with orthogonal *Q*, then we have

$$\begin{aligned} \langle \bar{G}_1, \bar{G}_2 \rangle _{\mathcal {X}} = {{\,\textrm{Tr}\,}}(\bar{G}^T_1 \bar{G}_2) = {{\,\textrm{Tr}\,}}(Q^T G_1^T G_2 Q)= {{\,\textrm{Tr}\,}}(G_1^T G_2 Q Q^T) = {{\,\textrm{Tr}\,}}(G_1^T G_2). \end{aligned}$$

It is easy to see that the norm induced by this inner product in any tangent space is the Frobenius norm, which we will denote throughout the paper as $\Vert \cdot \Vert :=\Vert \cdot \Vert _F$.

Exponential map. Given the Riemannian structure of ${{\,\textrm{Gr}\,}}(n,k)$, we can compute the exponential map at a point $\mathcal {X}$ as [1, Thm. 3.6]

4 $$\begin{aligned} \begin{aligned} {{\,\textrm{Exp}\,}}_{\mathcal {X}}: T_{\mathcal {X}} {{\,\textrm{Gr}\,}}(n,k)&\rightarrow {{\,\textrm{Gr}\,}}(n,k) \\ G&\mapsto {{\,\textrm{span}\,}}(\, X V \cos (\Sigma ) + U \sin (\Sigma ) \, ), \end{aligned} \end{aligned}$$

where $U \Sigma V^T$ is the *compact* SVD of *G* such that $\Sigma$ and *V* are square matrices.

The exponential map is invertible in the domain [7, Prop. 5.1]

5 $$\begin{aligned} \left\{ G \in T_{\mathcal {X}} {{\,\textrm{Gr}\,}}(n,k) :\Vert G \Vert _2 < \frac{\pi }{2} \right\} , \end{aligned}$$

where $\Vert G \Vert _2$ is the spectral norm of *G*. The inverse of the exponential map restricted to this domain is the logarithmic map, denoted by ${{\,\textrm{Log}\,}}$. Given two subspaces $\mathcal {X},\mathcal {Y}\in {{\,\textrm{Gr}\,}}(n,k)$, we have

6 $$\begin{aligned} {{\,\textrm{Log}\,}}_{\mathcal {X}}(\mathcal {Y}) = U {{\,\textrm{atan}\,}}(\widehat{\Sigma }) \, V^T, \end{aligned}$$

where $U \widehat{\Sigma }V^T = (I - X X^T) Y (X^T Y)^{-1}$ is again a compact SVD. This is well-defined if $X^T Y$ is invertible, which is guaranteed if all principal angles between $\mathcal {X}$ and $\mathcal {Y}$ are strictly less than $\pi / 2$ (see below). By taking $G = {{\,\textrm{Log}\,}}_{\mathcal {X}}(\mathcal {Y})$, we see that $\Sigma = {{\,\textrm{atan}\,}}(\widehat{\Sigma })$.

Principal angles. The Riemannian structure of the Grassmann manifold can be conveniently described by the notion of the principal angles between subspaces. Given two subspaces $\mathcal {X},\mathcal {Y} \in {{\,\textrm{Gr}\,}}(n,k)$, the principal angles between them are $0 \le \theta _1 \le \cdots \le \theta _k \le \pi /2$ obtained from the SVD

7 $$\begin{aligned} Y^T X=U_1 \cos \theta \ V_1^T \end{aligned}$$

where $U_1 \in \mathbb {R}^{k \times k}, V_1 \in \mathbb {R}^{k \times k}$ are orthogonal and the diagonal matrix $\cos \theta = {{\,\textrm{diag}\,}}(\cos \theta _1,...,\cos \theta _k)$. Notice that the definition of principal angles is indeed independent of the specific orthonormal representatives of the subspaces in question.

We can express the Riemannian logarithm using principal angles and the intrinsic distance induced by the Riemannian inner product discussed above is

8 $$\begin{aligned} {{\,\textrm{dist}\,}}(\mathcal {X},\mathcal {Y)}=\Vert {{\,\textrm{Log}\,}}_{\mathcal {X}} (\mathcal {Y}) \Vert = \Vert {{\,\textrm{Log}\,}}_{\mathcal {Y}} (\mathcal {X}) \Vert =\sqrt{\theta _1^2+...+\theta _k^2}=\Vert \theta \Vert _2, \end{aligned}$$

where $\theta =(\theta _1, \ldots ,\theta _k)^T$. For more details on these facts, the reader can refer to section 4.3 in [12] (arc length distance).

If $X \in \mathbb {R}^{n \times k}$ is an arbitrary matrix with orthonormal columns, then, generically, these columns will not be exactly orthogonal to the *k* leading eigenvectors $v_1, \ldots , v_k$ of *A*. Thus, we have with probability one that the principal angles between $\mathcal {X}$ and the space of *k* leading eigenvectors satisfy $0 \le \theta _1 \le \cdots \le \theta _k < \pi /2$.

Curvature. We can compute exactly the sectional curvatures in ${{\,\textrm{Gr}\,}}(n,k)$, but for our purposes we only need that they are everywhere non-negative [7, 33]. This means that the geodesics on the Grassmann manifold spread more slowly than in Euclidean space. This is consequence of the famous Toponogov’s theorem (see [11]) that we state here in the form of the following technical lemma, which will be important in our convergence analysis.

### Lemma 3.1

Let $\mathcal {X}, \mathcal {Y}, \mathcal {Z} \in {{\,\textrm{Gr}\,}}(n,k)$, such that

$$\max \{ \text {dist}(\mathcal {X}, \mathcal {Z}), \text {dist} (\mathcal {Y}, \mathcal {Z}) \} < \frac{\pi }{2}. $$

Then

$$\begin{aligned} \text {dist}(\mathcal {X}, \mathcal {Y}) \le \Vert {{\,\textrm{Log}\,}}_\mathcal {Z}(\mathcal {X})-{{\,\textrm{Log}\,}}_\mathcal {Z}(\mathcal {Y}) \Vert . \end{aligned}$$

### Lemma 3.2

(Law of cosines) Let $\mathcal {X},\mathcal {Y},\mathcal {Z}$ as in Lemma 3.1. Then

$$\begin{aligned} {{\,\textrm{dist}\,}}^2(\mathcal {X}, \mathcal {Y}) \le {{\,\textrm{dist}\,}}^2(\mathcal {Z}, \mathcal {X})+ {{\,\textrm{dist}\,}}^2(\mathcal {Z}, \mathcal {Y})-2 \langle {{\,\textrm{Log}\,}}_{\mathcal {Z}} (\mathcal {X}), {{\,\textrm{Log}\,}}_{\mathcal {Z}} (\mathcal {Y}) \rangle . \end{aligned}$$

### Proof

Apply Lemma 3.1 and expand $\Vert {{\,\textrm{Log}\,}}_\mathcal {Z}(\mathcal {X})-{{\,\textrm{Log}\,}}_\mathcal {Z}(\mathcal {Y}) \Vert ^2$. $\square$

Block Rayleigh quotient. Our objective function for minimization is the block version of the Rayleigh quotient:

$$\begin{aligned} f(\mathcal {X}) = -{{\,\textrm{Tr}\,}}(X^TAX) \text { where}\,\mathcal {X} = {{\,\textrm{span}\,}}(X) \in {{\,\textrm{Gr}\,}}(n,k)\hbox { s.t. }X^T X = I_k. \end{aligned}$$

This function has $$\mathcal {V}_{\alpha }={{\,\textrm{span}\,}}(\begin{bmatrix} v_1 \hspace{2mm} \cdots \hspace{2mm} v_k \end{bmatrix})$$ as global minimizer. This minimizer is unique on ${{\,\textrm{Gr}\,}}(n,k)$ if and only if $\delta >0$.

Given any differentiable function $f:{{\,\textrm{Gr}\,}}(n,k) \rightarrow \mathbb {R}$, we can define its Riemannian gradient as the vector field that satisfies

$$\begin{aligned} df(\mathcal {X})(G) = \langle {{\,\textrm{grad}\,}}f(\mathcal {X}), G \rangle _{\mathcal {X}}, \hspace{1mm} \text {for any} \hspace{1mm} G \in T_{\mathcal {X}} {{\,\textrm{Gr}\,}}(n,k). \end{aligned}$$

For a given representative *X* of $\mathcal {X}$, the Riemannian gradient of the block Rayleigh quotient satisfies

$$\begin{aligned} {{\,\textrm{grad}\,}}f(\mathcal {X}) = -2(I-X X^T) A X. \end{aligned}$$

Using the notions of the Riemannian gradient and Levi-Civita connection, we can define also a Riemannian notion of Hessian. For the block Rayleigh quotient *f*, the Riemannian Hessian ${{\,\textrm{Hess}\,}}f$ evaluated as bilinear form satisfies

9 $$\begin{aligned} {{\,\textrm{Hess}\,}}f(\mathcal {X})[{G},{G}] = 2 \langle G, G X^T A X - A G \rangle , \end{aligned}$$

for $G \in T_{\mathcal {X}} {{\,\textrm{Gr}\,}}(n,k)$; see [12, §4.4] or [2, §6.4.2].

## Convexity-like Properties of the Block Rayleigh quotient

We now prove the new analytic properties of the block Rayleigh quotient $f(\mathcal {X})=-{{\,\textrm{Tr}\,}}(X^T A X)$. These are important in their own right but will also be used later for the convergence of the Riemannian steepest descent method.

### Smoothness

A $C^2$ function defined on the Grassmann manifold is called $\gamma$-smooth if the maximum eigenvalue of its Riemannian Hessian is everywhere upper bounded by a positive constant $\gamma$. This is true for the block Rayleigh quotient, as we show in the next proposition:

#### Proposition 1

(Smoothness) The eigenvalues of the Riemannian Hessian of *f* on ${{\,\textrm{Gr}\,}}(n,k)$ are upper bounded by $\gamma := 2 (\lambda _1 - \lambda _n)$.

#### Proof

Let *G* be a tangent vector of ${{\,\textrm{Gr}\,}}(n,k)$ at *X*. Then the Riemannian Hessian satisfies (see (9))

$$\begin{aligned} \tfrac{1}{2} {{\,\textrm{Hess}\,}}f(\mathcal {X})[G,G] = {{\,\textrm{Tr}\,}}(G^T G X^T A X) - {{\,\textrm{Tr}\,}}(A G G^T). \end{aligned}$$

Since $A, X^T A X, G G^T$, and $G^T G$ are all symmetric and positive semi-definite matrices, standard trace inequality (see, e.g, [18, Thm. 4.3.53]) gives

$$\begin{aligned} {{\,\textrm{Hess}\,}}f(\mathcal {X})[G,G] \le 2 (\lambda _{\max }(X^T A X)- \lambda _{\min } (A)) \Vert G \Vert ^2. \end{aligned}$$

Since *X* has orthonormal columns, $\lambda _{\max }(X^T A X) \le \lambda _{\max }( A )$; see, e.g., [18, Cor. 4.3.37]. The proof is now complete with the definition of $\lambda _1$ and $\lambda _n$. $\square$

The result in Prop. 1 is tight: Choosing $X = V_\alpha$ and $G = v_n e_1^T$, it is readily verified that the upper bound is attained. From now on, we refer to $\gamma$ as the specific value $2(\lambda _1-\lambda _n)$. This value also features in a useful upper bound for the spectral norm of the gradient. This bound is independent of $\mathcal {X}$:

#### Lemma 4.1

For all $\mathcal {X} \in {{\,\textrm{Gr}\,}}(n,k)$ and $\gamma =2(\lambda _1-\lambda _n)$, the Riemannian gradient of *f* satisfies

$$\begin{aligned} \Vert {{\,\textrm{grad}\,}}f(\mathcal {X}) \Vert _2 \le \frac{\gamma }{2}. \end{aligned}$$

#### Proof

Since *X* has orthonormal columns, we can complete it to the orthogonal matrix $$Q = \begin{bmatrix} X&X_\perp \end{bmatrix}$$. Hence, $\Vert {{\,\textrm{grad}\,}}f(\mathcal {X}) \Vert _2 = \Vert 2 (I - XX^T)AX \Vert _2 = 2 \Vert X_\perp ^T A X \Vert _2$. The result now follows directly from [22, Thm. 2] since *A* is real symmetric and the definition of $\gamma = 2(\lambda _1 - \lambda _n)$. $\square$

By the second-order Taylor expansion of *f* (see, e.g., [8], Corollary 10.54) it is easy to see that Proposition 1 implies

10 $$\begin{aligned} f(\mathcal {X}) \le f(\mathcal {Y})+ \langle {{\,\textrm{grad}\,}}f(\mathcal {Y}), {{\,\textrm{Log}\,}}_{\mathcal {Y}} (\mathcal {X}) \rangle +\frac{\gamma }{2} {{\,\textrm{dist}\,}}^2(\mathcal {X},\mathcal {Y}), \end{aligned}$$

for any $\mathcal {X}, \mathcal {Y} \in {{\,\textrm{Gr}\,}}(n,k)$ such that ${{\,\textrm{Log}\,}}_{\mathcal {X}}(\mathcal {Y})$ is well-defined.

As in the introduction, denote the global minimum of *f* by $f^*$ which is attained at $\mathcal {V}_\alpha \in {{\,\textrm{Gr}\,}}(n,k)$. Inequality (10) leads to the following lemma:

#### Lemma 4.2

For any $\mathcal {X} \in {{\,\textrm{Gr}\,}}(n,k)$ and $\gamma =2(\lambda _1-\lambda _n)$, we have

$$\begin{aligned} f(\mathcal {X})-f^* \ge \frac{1}{2 \gamma } \Vert {{\,\textrm{grad}\,}}f(\mathcal {X}) \Vert ^2. \end{aligned}$$

#### Proof

Since $f^*$ is a global minimum of *f*, we have from (10) that

$$\begin{aligned} f^* \le f(\mathcal {X}) \le f(\mathcal {Y})+\langle \text {grad}f(\mathcal {Y}),{{\,\textrm{Log}\,}}_{\mathcal {Y}}(\mathcal {X}) \rangle +\frac{\gamma }{2} \Vert {{\,\textrm{Log}\,}}_{\mathcal {Y}}(\mathcal {X}) \Vert ^2, \end{aligned}$$

for any $\mathcal {X}, \mathcal {Y} \in {{\,\textrm{Gr}\,}}(n,k)$ such that ${{\,\textrm{Log}\,}}_{\mathcal {X}}(\mathcal {Y})$ is well-defined.

We set $\mathcal {X}:={{\,\textrm{Exp}\,}}_{\mathcal {Y}} \left( -\frac{1}{\gamma } \text {grad}f(\mathcal {Y})\right)$. By Lemma 4.1, we have that $\left\| -\frac{1}{\gamma } \text {grad}f(\mathcal {Y}) \right\| _2<\frac{\pi }{2}$ and by equation (5) we have that ${{\,\textrm{Log}\,}}_{\mathcal {Y}}(\mathcal {X})$ is well-defined and equal to $-\frac{1}{\gamma } \text {grad}f(\mathcal {Y})$. Then,

the right hand side of the initial inequality becomes

$$\begin{aligned} f^* \le f(\mathcal {Y})-\frac{1}{\gamma } \Vert \text {grad}f(\mathcal {Y})\Vert ^2+ \frac{1}{2\gamma } \Vert \text {grad}f(\mathcal {Y})\Vert ^2 = f(\mathcal {Y})-\frac{1}{2 \gamma } \Vert \text {grad}f(\mathcal {Y})\Vert ^2. \end{aligned}$$

Rearranging the last inequality and substituting $\mathcal {Y}=\mathcal {X}$, we get the desired result. $\square$

### Weak-Quasi-Convexity and Quadratic Growth

We now turn our interest in the convexity properties of the block Rayleigh quotient function. We start by proving a property which is known in the literature as *quadratic growth*.

#### Proposition 2

(Quadratic growth) Let $0 \le \theta _1 \le \cdots \le \theta _k < \pi /2$ be the principal angles between the subspaces $\mathcal {X}$ and $\mathcal {V}_\alpha$. The function *f* satisfies

$$f(\mathcal {X})-f^* \ge c_Q \, \delta \, {{\,\textrm{dist}\,}}^2(\mathcal {X},\mathcal {V_{\alpha }})$$

where $$c_Q = 4/\pi ^2 > 0.4$$.

#### Proof

The spectral decomposition of $A = V_{\alpha } \Lambda _{\alpha }V_{\alpha }^T + V_{\beta } \Lambda _{\beta } V_{\beta }^T$ implies

11 $$\begin{aligned} X^T AX = X^T V_{\alpha } \Lambda _{\alpha }V_{\alpha }^T X+ X^T V_{\beta } \Lambda _{\beta } V_{\beta }^T X. \end{aligned}$$

Since $f(\mathcal {X}) = -{{\,\textrm{Tr}\,}}(X^TAX)$, we have

$$\begin{aligned} f(\mathcal {X})-f^*&= {{\,\textrm{Tr}\,}}(\Lambda _{\alpha })-{{\,\textrm{Tr}\,}}(X^T V_{\alpha } \Lambda _{\alpha }V_{\alpha }^T X)-{{\,\textrm{Tr}\,}}(X^T V_{\beta } \Lambda _{\beta } V_{\beta }^T X) \\  &= {{\,\textrm{Tr}\,}}(\Lambda _{\alpha })-{{\,\textrm{Tr}\,}}( \Lambda _{\alpha }V_{\alpha }^T X X^T V_{\alpha })-{{\,\textrm{Tr}\,}}( \Lambda _{\beta } V_{\beta }^T X X^T V_{\beta }) \\  &= {{\,\textrm{Tr}\,}}(\Lambda _{\alpha } (I_k- V_{\alpha }^T X X^T V_{\alpha })) - {{\,\textrm{Tr}\,}}(\Lambda _{\beta } V_{\beta }^T X X^T V_{\beta }). \end{aligned}$$

From the definition (7) of the principal angles between *X* and $V_{\alpha }$, we recall that

12 $$\begin{aligned} V_{\alpha }^T X=U_1 \cos \theta \, V_1^T, \end{aligned}$$

where $\cos \theta = {{\,\textrm{diag}\,}}(\cos \theta _1, \ldots , \cos \theta _k)$ is a diagonal matrix and $U_1, V_1$ are orthogonal matrices. Plugging this equality in, we get that the *j*th eigenvalue of the matrix $I_k- V_{\alpha }^T X X^T V_{\alpha }$ is equal to $1-\cos ^2\theta _j = \sin ^2 \theta _j \ge 0$. Thus, by standard trace inequality for symmetric and positive definite matrices (see, e.g., [18, Thm. 4.3.53]), the first summand above satisfies

$$\begin{aligned} {{\,\textrm{Tr}\,}}(\Lambda _{\alpha } (I_k- V_{\alpha }^T X X^T V_{\alpha })) \ge \lambda _{k} \sum _{j=1}^k \sin ^2 \theta _j. \end{aligned}$$

The matrix $V_{\beta }^T X X^T V_{\beta }$ has the same non-zero eigenvalues with the same multiplicity as the matrix

$$\begin{aligned} X^T V_{\beta } V_{\beta }^T X = I_k-V_1 \cos ^2 \theta \, V_1^T = V_1 \sin ^2 \theta \, V_1^T \end{aligned}$$

where we used $V_{\beta } V_{\beta }^T= I_n - V_{\alpha } V_{\alpha }^T$ and the SVD of $V_{\alpha }^T X$. Thus the *j*th eigenvalue of $V_{\beta }^T X X^T V_{\beta }$ is $\sin ^2 \theta _j \ge 0$. By trace inequality again, the second summand therefore satisfies

$$\begin{aligned} {{\,\textrm{Tr}\,}}(\Lambda _{\beta } V_{\beta }^T X X^T V_{\beta }) \le \lambda _{k+1} \sum _{j=1}^k \sin ^2 \theta _j. \end{aligned}$$

Putting both bounds together, we get

$$\begin{aligned} f(\mathcal {X})-f^* \ge (\lambda _k - \lambda _{k+1}) \sum _{j=1}^k \sin ^2 \theta _j \ge \delta \sum _{j=1}^k \frac{4}{\pi ^2}\theta _j^2 \end{aligned}$$

and the proof is complete by the definition (8) of ${{\,\textrm{dist}\,}}$. $\square$

We say that *f* is geodesically convex if for all $\mathcal {X}$ and $\mathcal {Y}$ in a suitable region it holds

$$ f(\mathcal {X})-f(\mathcal {Y}) \le \langle \text {grad}f(\mathcal {X}), -{{\,\textrm{Log}\,}}_{\mathcal {X}}(\mathcal {Y}) \rangle .$$

This generalizes the classical convexity of differentiable functions on $\mathbb {R}^n$ to manifolds by taking the logarithmic map instead of the difference $\mathcal {X}-\mathcal {Y}$.

In Appendix A, we prove that our objective function *f* is only geodesically convex in a small neighbourhood of size ${\mathcal {O}}(\sqrt{\delta })$ around the minimizer $\mathcal {V}_\alpha$. Fortunately, our key result of this section shows that *f* satisfies a much weaker notion of geodesic convexity, known in the literature as *weak-quasi-convexity*, that does not depend on the eigengap $\delta$.

We first need the following lemma which is the general version of the CS decomposition but applied to our setting of square blocks.

#### Lemma 4.3

Let $X,Y \in \mathbb {R}^{n \times k}$ be such that $X^T X = Y^T Y = I_k$ with $k < n$. Choose $X_\perp , Y_\perp \in \mathbb {R}^{n \times (n-k)}$ such that $X_\perp ^T X_\perp = Y_\perp ^T Y_\perp = I_{n-k}$ and ${{\,\textrm{span}\,}}(X_\perp ) = {{\,\textrm{span}\,}}(X)^\perp$, ${{\,\textrm{span}\,}}(Y_\perp ) = {{\,\textrm{span}\,}}(Y)^\perp$. Then there exist $0 \le r,s \le k$ such that

$$\begin{aligned} Y^T X&= U_1 \begin{bmatrix}I_r \\   &  C_s \\   &  &  O_{p \times p} \end{bmatrix} V_1^T,&Y^T X_\perp&= U_1 \begin{bmatrix}O_{r \times m} \\   &  S_s \\   &  &  I_{p} \end{bmatrix} V_2^T \\ Y_\perp ^T X&= U_2 \begin{bmatrix}O_{m \times r} \\   &  S_s \\   &  &  I_{p} \end{bmatrix} V_1^T,&Y_\perp ^T X_\perp&= U_2 \begin{bmatrix}-I_{m} \\   &  -C_s \\   &  &  O_{p \times p} \end{bmatrix} V_2^T \end{aligned}$$

with $p=k-r-s$ and $m = n - 2k +r$, and we have

- orthogonal matrices $U_1, V_1$ of size *k* and $U_2, V_2$ of size $n-k$;
- identity matrices $I_q$ of size *q*;
- zero matrices $O_{q \times t}$ of size $q \times t$;
- diagonal matrices $C_s = {{\,\textrm{diag}\,}}(\alpha _1, \ldots , \alpha _s)$ and $S_s = {{\,\textrm{diag}\,}}(\beta _1, \ldots , \beta _s)$ such that $1> \alpha _1 \ge \cdots \ge \alpha _s > 0$, $0< \beta _1 \le \cdots \le \beta _s < 1$ and $C_s^2 + S_s^2 = I_s$.

#### Proof

Since $$\begin{bmatrix} X&X_\perp \end{bmatrix}$$ and $$\begin{bmatrix} Y&Y_\perp \end{bmatrix}$$ are orthogonal, the result follows directly from the CS decomposition of the orthogonal matrix $$P = \begin{bmatrix} Y&Y_\perp \end{bmatrix}^T \begin{bmatrix} X&X_\perp \end{bmatrix}$$; see the Theorem of §4 in [29]. $\square$

Observe that the matrix ${{\,\textrm{diag}\,}}(I_r,C_s,O_{p \times p})$ in this lemma corresponds to the matrix $\cos (\theta )$ in (7) with $\theta$ the vector of principal angles $0 \le \theta _1 \le \cdots \le \theta _k \le \pi /2$ between ${{\,\textrm{span}\,}}(X)$ and ${{\,\textrm{span}\,}}(Y)$. However, the lemma explicitly splits off the angles that are zero and $\pi /2$ so that it can formulate the related decompositions for $Y^T X_\perp , Y_\perp ^T X,$ and $Y_\perp ^T X_\perp$ with $C_s$ and $S_s$.

We are now ready to state our weak quasi-convexity result. In the statement of the proposition below (and throughout this paper), we use the convention that $\frac{0}{\tan 0} = 1$.

#### Proposition 3

(Weak-quasi-convexity) Let $0 \le \theta _1 \le \cdots \le \theta _k < \pi /2$ be the principal angles between the subspaces $\mathcal {X}$ and $\mathcal {V}_\alpha$. Then, *f* satisfies

$$2 a(\mathcal {X}) \, (f(\mathcal {X})-f^*) \le \langle \text {grad}f(\mathcal {X}), -{{\,\textrm{Log}\,}}_{\mathcal {X}}(\mathcal {V_{\alpha })} \rangle $$

with $$a(\mathcal {X}):= \theta _k / \tan \theta _k$$.

#### Proof

Take *X* and $V_\alpha$ matrices with orthonormal columns such that $\mathcal {X} = {{\,\textrm{span}\,}}(X)$ and $\mathcal {V}_\alpha = {{\,\textrm{span}\,}}(V_\alpha )$. Since $\theta _k < \pi / 2$, we know that $p=0$ in Lemma 4.3 and thus $s = k - r$ with *r* the number of principal angles that are equal to zero. Choosing a matrix $X_\perp$ with orthonormal columns such that ${{\,\textrm{span}\,}}(X_\perp ) = {{\,\textrm{span}\,}}(X)^\perp$, we therefore get from Lemma 4.3 that there exist orthogonal matrices $U_1,V_1$ of size *k* and $V_2$ of size $n-k$ such that

13 $$\begin{aligned} V_\alpha ^T X&= U_1 \begin{bmatrix}I_r \\   &  C_{k-r} \end{bmatrix} V_1^T,&V_\alpha ^T X_\perp&= U_1 \begin{bmatrix}O_{r \times m} \\ &  S_{k-r} \end{bmatrix} V_2^T. \end{aligned}$$

Comparing with (7), we deduce that $C_{k-r} = {{\,\textrm{diag}\,}}(\cos \theta _{r+1}, \ldots , \cos \theta _k)$ and $S_{k-r} = {{\,\textrm{diag}\,}}(\sin \theta _{r+1}, \ldots , \sin \theta _k)$ since $C_{k-r}^2 + S_{k-r}^2 = I$.

We recall from (6) that

14 $$\begin{aligned} {{\,\textrm{Log}\,}}_{\mathcal {X}}(\mathcal {V}_{\alpha }) = U {{\,\textrm{atan}\,}}(\Sigma ) V^T, \end{aligned}$$

where $U \Sigma V^T=(I_n - X X^T) V_{\alpha } (X^T V_{\alpha })^{-1}=:M$ is a compact SVD (without the requirement that the diagonal of $\Sigma$ is non-increasing). Using $X_\perp$ from above, we can also write $M = X_\perp X_\perp ^T V_{\alpha } (X^T V_{\alpha })^{-1}$. Substituting (13) and using that $U_1$ and $V_1$ are orthogonal gives

$$\begin{aligned} M = X_\perp V_2 \begin{bmatrix}O_{m \times r} \\   &  S_{k-r} C_{k-r}^{-1} \end{bmatrix} V_1^T = X_\perp \tilde{V}_2 \begin{bmatrix}O_{r \times r} \\   &  S_{k-r} C_{k-r}^{-1} \end{bmatrix} V_1^T, \end{aligned}$$

where $\tilde{V}_2 \in \mathbb {R}^{(n-k) \times k}$ contains the last *k* columns of $V_2$ in order. Note that this reformulation of the SVD of *M* holds always, regardless of the relationship between *m* and *r*. If $m \ge r$, the matrix $$\begin{bmatrix}O_{m \times r} \\   &  S_{k-r} C_{k-r}^{-1} \end{bmatrix}$$ has its first $m-r$ rows equal to 0, thus we can cut the first $m-r$ columns of $V_2$, since they do not contribute to the product. This yields a matrix $\tilde{V}_2$ with $n-k$ rows and $n-k-m+r=k$ of the last columns of $V_2$. If $m<r$, then the first $r-m$ columns of $$\begin{bmatrix}O_{m \times r} \\   &  S_{k-r} C_{k-r}^{-1} \end{bmatrix}$$ are 0 and now we can add $r-m$ columns in the beginning of the matrix $V_2$ that keep the derived matrix orthonormal. This again yields a matrix $\tilde{V}_2$ with $n-k$ rows and $n-k+r-m=k$ columns. Since the matrix $$\begin{bmatrix}O_{r \times r} \\   &  S_{k-r} C_{k-r}^{-1} \end{bmatrix}$$ occurs by adding $r-m$ zero rows at the beginning of $$\begin{bmatrix}O_{m \times r} \\   &  S_{k-r} C_{k-r}^{-1} \end{bmatrix}$$, the product does not change.

Since $\theta _1 = \cdots = \theta _r = 0$, we can therefore formulate the compact SVD of *M* using the vector $\theta$ of all principal angles as follows:

$$\begin{aligned} M = U \Sigma V^T \quad \text {with}\,U = X_\perp \tilde{V}_2, \ \Sigma = \tan (\theta ), \ V = V_1. \end{aligned}$$

Hence from (14) we get directly that

15 $$\begin{aligned} {{\,\textrm{Log}\,}}_{\mathcal {X}}(\mathcal {V}_{\alpha }) = X_\perp \tilde{V}_2\, \theta \, V_1^T, \end{aligned}$$

where $\theta$ is a diagonal matrix.

We now claim that (15) also satisfies

16 $$\begin{aligned} {{\,\textrm{Log}\,}}_{\mathcal {X}}(\mathcal {V}_{\alpha }) = X_\perp X_\perp ^T V_\alpha U_1 \frac{\theta }{\sin \theta } V_1^T, \end{aligned}$$

where $\frac{\theta }{\sin \theta }$ is a diagonal matrix for which $\frac{0}{\sin 0} = 1$. Indeed, recalling that $\theta _1 = \cdots = \theta _r = 0$ and using the identities

$$\begin{aligned} X_\perp ^T V_\alpha = \tilde{V}_2 \begin{bmatrix}O_{r \times r} \\   &  S_{k-r} \end{bmatrix} U_1^T, \quad \frac{\theta }{\sin \theta } = \begin{bmatrix}I_{r} \\   &  S_{k-r}^{-1} \end{bmatrix} \begin{bmatrix}I_{r} \\   &  T_{k-r} \end{bmatrix} \end{aligned}$$

where $T_{k-r}={{\,\textrm{diag}\,}}(\theta _{r+1}, \ldots , \theta _k)$, we obtain

$$\begin{aligned} \text {rhs of~(16)}&= X_\perp \tilde{V}_2 \begin{bmatrix}O_{r \times r} \\   &  S_{k-r} \end{bmatrix} \begin{bmatrix}I_{r} \\   &  S_{k-r}^{-1} \end{bmatrix} \begin{bmatrix}I_{r} \\   &  T_{k-r} \end{bmatrix} \, V_1^T \\  &= X_\perp \tilde{V}_2 \begin{bmatrix} O_{r \times r} \\   &  T_{k-r} \end{bmatrix} \, V_1^T = X_\perp \tilde{V}_2 \, \theta \, V_1^T = \text {rhs of~(15)}. \end{aligned}$$

Next, we work out

$$\begin{aligned} s:= \langle {{\,\textrm{grad}\,}}f(\mathcal {X}), -{{\,\textrm{Log}\,}}_{\mathcal {X}}(\mathcal {V_{\alpha })} \rangle . \end{aligned}$$

Since ${{\,\textrm{grad}\,}}f(\mathcal {X})$ and ${{\,\textrm{Log}\,}}_{\mathcal {X}}(\mathcal {V}_{\alpha })$, respectively, give tangent vectors for the same representative *X* of $\mathcal {X}$, the inner product above is the trace of the corresponding matrix representations. Using (16) with $I-XX^T = X_\perp X_\perp ^T$, we therefore get

$$\begin{aligned} s&= 2 \Big \langle (I-XX^T)AX, (I-XX^T) V_\alpha U_1 \frac{\theta }{\sin (\theta )} V_1^T \Big \rangle \\&= 2 {{\,\textrm{Tr}\,}}\Big ( \frac{\theta }{\sin (\theta )} U_1^T V_\alpha ^T (I-XX^T) AX V_1 \Big ). \end{aligned}$$

Since $AV_\alpha = V_\alpha \Lambda _\alpha$, we can simplify

17 $$\begin{aligned} V_\alpha ^T (I-XX^T) AX = \Lambda _\alpha V_\alpha ^T X - V_\alpha ^T XX^T AX. \end{aligned}$$

Substituting in the expression above and using that $V_{\alpha }^T X=U_1 \cos \theta \, V_1^T$, we get

$$\begin{aligned} \frac{1}{2} s&= {{\,\textrm{Tr}\,}}\Big ( \frac{\theta }{\sin (\theta )} U_1^T \Lambda _\alpha U_1 \cos (\theta ) \Big ) - {{\,\textrm{Tr}\,}}\Big ( \frac{\theta }{\sin (\theta )} \cos (\theta ) V_1^T X^T AX V_1 \Big ) \\&= {{\,\textrm{Tr}\,}}\Big ( \frac{\theta }{\tan (\theta )} \Big ( U_1^T \Lambda _\alpha U_1 - V_1^T X^T AX V_1 \Big ) \Big ), \end{aligned}$$

with the convention $\frac{0}{\tan 0} = 1$.

Denote the symmetric matrix

18 $$\begin{aligned} S:= U_1^T \Lambda _\alpha U_1 - V_1^T X^T AX V_1. \end{aligned}$$

We show below that all diagonal entries $S_{11}, \ldots , S_{kk}$ of *S* are nonnegative. Hence, by diagonality of the matrix $\tfrac{\theta }{\tan (\theta )}$, we obtain

$$\begin{aligned} \frac{1}{2} s&= \sum _j \frac{\theta _j}{\tan \theta _j} \, S_{jj} \ge \min _j \frac{\theta _j}{\tan \theta _j} \, {{\,\textrm{Tr}\,}}(S) = \frac{\theta _k}{\tan \theta _k} \, \Big [ {{\,\textrm{Tr}\,}}( \Lambda _{\alpha }) - {{\,\textrm{Tr}\,}}( X^T AX ) \Big ] \end{aligned}$$

since $U_1$ and $V_1$ are orthogonal matrices. We recover the desired result after substituting $f(\mathcal {X}) = -{{\,\textrm{Tr}\,}}(X^T A X)$ and $f_* = -{{\,\textrm{Tr}\,}}(V_\alpha ^T A V_\alpha ) = -{{\,\textrm{Tr}\,}}(\Lambda _\alpha )$.

It remains to show that $S_{jj} \ge 0$ for $j=1,\ldots , k$. Since ${{\,\textrm{span}\,}}(V_\beta ) = {{\,\textrm{span}\,}}(V_\alpha )^\perp$, Lemma 4.3 gives us in addition to (13) also

19 $$\begin{aligned} V_\beta ^T X = U_2 \begin{bmatrix} O_{m \times r} \\   &  S_{k-r} \end{bmatrix} V_1^T = \tilde{U}_2 \sin \theta \, V_1^T, \end{aligned}$$

where $\tilde{U}_2 \in \mathbb {R}^{(n-k) \times k}$ contains the last *k* columns of the orthogonal matrix $U_2$ in order. A short calculation using (11) then shows that (18) satisfies

$$\begin{aligned} S= U_1^T \Lambda _\alpha U_1 - \cos \theta \, U_1^T \Lambda _\alpha U_1 \cos \theta - \sin \theta \, \tilde{U}_2^T \Lambda _\beta \tilde{U}_2 \sin \theta \end{aligned}$$

with diagonal elements

$$\begin{aligned} S_{jj} = \sin ^2\theta _j \, (U_1^T \Lambda _\alpha U_1 -\tilde{U}_2^T \Lambda _\beta \tilde{U}_2)_{jj}. \end{aligned}$$

Since $U_1$ and $\tilde{U}_2$ have orthonormal columns, we obtain

$$\begin{aligned} \lambda _{\min } (U_1^T \Lambda _\alpha U_1) \ge \lambda _{\min } (\Lambda _\alpha ) = \lambda _k, \quad \lambda _{\max }(\tilde{U}_2^T \Lambda _\beta \tilde{U}_2) \le \lambda _{\max }(\Lambda _\beta ) = \lambda _{k+1}, \end{aligned}$$

from which we get with Weyl’s inequality that

$$\begin{aligned} \lambda _{\min } (U_1^T \Lambda _\alpha U_1 - \tilde{U}_2^T \Lambda _\beta \tilde{U}_2) \ge \lambda _{\min } (U_1^T \Lambda _\alpha U_1) - \lambda _{\max }(\tilde{U}_2^T \Lambda _\beta \tilde{U}_2) \ge \lambda _k - \lambda _{k+1} \ge 0. \end{aligned}$$

Hence, the matrix

20 $$\begin{aligned} U_1^T \Lambda _\alpha U_1 - \tilde{U}_2^T \Lambda _\beta \tilde{U}_2 \end{aligned}$$

is symmetric and positive semi-definite. Its diagonal entries, and thus also $S_{jj}$, are therefore nonnegative. $\square$

We now arrive at a useful property of *f* that will later allow us to analyze the convergence of Riemannian steepest descent. It is a *weaker version of strong geodesic convexity* and can be proved easily using quadratic growth and weak-quasi-convexity.

#### Theorem 4.1

(Weak-strong convexity) Let $0 \le \theta _1 \le \cdots \le \theta _k < \pi /2$ be the principal angles between the subspaces $\mathcal {X}$ and $\mathcal {V}_\alpha$. Then, *f* satisfies

$$\begin{aligned} f(\mathcal {X})-f^* \le \frac{1}{a(\mathcal {X})} \langle {{\,\textrm{grad}\,}}f(\mathcal {X}), -{{\,\textrm{Log}\,}}_{\mathcal {X}}(\mathcal {V}_{\alpha }) \rangle - c_Q \delta \, {{\,\textrm{dist}\,}}^2 (\mathcal {X},\mathcal {V}_{\alpha }) \end{aligned}$$

with $$a(\mathcal {X})= \theta _k / \tan \theta _k >0$$, $$c_Q = 4/\pi ^2 > 0.4$$, and $$\delta = \lambda _k - \lambda _{k+1} \ge 0$$.

#### Proof

Combining Propositions 2 and 3 leads to

$$\begin{aligned} c_Q \delta \, {{\,\textrm{dist}\,}}^2(\mathcal {X},\mathcal {V}_{\alpha }) \le f(\mathcal {X})-f^* \le \frac{1}{2 a(\mathcal {X})} \langle {{\,\textrm{grad}\,}}f(\mathcal {X}), -{{\,\textrm{Log}\,}}_{\mathcal {X}}(\mathcal {V}_{\alpha }) \rangle . \end{aligned}$$

At the same time, Proposition 3 also implies

$$\begin{aligned} f(\mathcal {X})-f^*&\le \frac{1}{2 a(\mathcal {X})} \langle {{\,\textrm{grad}\,}}f(\mathcal {X}), -{{\,\textrm{Log}\,}}_{\mathcal {X}}(\mathcal {V}_\alpha ) \rangle - c_Q \delta \, {{\,\textrm{dist}\,}}^2(\mathcal {X},\mathcal {V}_\alpha ) \\  &\qquad + c_Q \delta \, {{\,\textrm{dist}\,}}^2(\mathcal {X},\mathcal {V}_\alpha ). \end{aligned}$$

Using the first inequality to bound the last term of the right hand side, we recover the desired result. $\square$

#### Remark 4.1

Theorem 4.1 is also valid when the eigengap $\delta = 0$. In that case, $\mathcal {V}_{\alpha }$ is *any subspace spanned by* *k* *leading eigenvectors of* *A* and the theorem (almost) reduces to Proposition 3 (up to a scalar 2).

While not needed for our convergence proof, the next result is of independent interest and shows that *f* is gradient dominated in the Riemannian sense when the eigengap $\delta$ is strictly positive. This property is the *Riemannian version of the Polyak–Łojasiewicz inequality* and generalizes a result by [34] for the Rayleigh quotient on the sphere.

#### Proposition 4

(Gradient dominance) The function *f* satisfies

$$\begin{aligned} \Vert \text {grad}f(\mathcal {X}) \Vert ^2 \ge 4 \, c_Q \, \delta \, a^2(\mathcal {X}) (f(\mathcal {X})-f^*) \end{aligned}$$

for all subspaces $\mathcal {X}$ that have a largest principal angle $<\pi /2$ with $\mathcal {V}_\alpha$.

#### Proof

We assume that $\delta > 0$ since otherwise the statement is trivially true. By Theorem 4.1, we have

$$\begin{aligned} f(\mathcal {X})-f^* \le \frac{1}{a(\mathcal {X})} \langle {{\,\textrm{grad}\,}}f(\mathcal {X}), -{{\,\textrm{Log}\,}}_{\mathcal {X}}(\mathcal {V}_{\alpha }) \rangle - c_Q \delta {{\,\textrm{dist}\,}}^2 (\mathcal {X}, \mathcal {V}_{\alpha }). \end{aligned}$$

Since $\langle G_1, G_2 \rangle \le \frac{\rho }{2} \Vert G_1\Vert ^2 + \frac{1}{2 \rho } \Vert G_2 \Vert ^2$ for all matrices $G_1, G_2$ and $\rho >0$, we can write (for any $\rho > 0$) that

$$\begin{aligned} \langle {{\,\textrm{grad}\,}}f(\mathcal {X}), -{{\,\textrm{Log}\,}}_{\mathcal {X}}(\mathcal {V}_{\alpha }) \rangle \le \frac{\rho }{2} \Vert \text {grad}f(\mathcal {X}) \Vert ^2 + \frac{1}{2 \rho } \Vert {{\,\textrm{Log}\,}}_{\mathcal {X}}(\mathcal {V}_{\alpha }) \Vert ^2. \end{aligned}$$

Using that ${{\,\textrm{dist}\,}}(\mathcal {X}, \mathcal {V}_{\alpha })= \Vert {{\,\textrm{Log}\,}}_{\mathcal {X}}(\mathcal {V}_{\alpha }) \Vert$ and choosing $\rho =1/(2 c_Q \delta a(\mathcal {X}))$, we get the desired result. $\square$

## Convergence of Riemannian Steepest Descent

We now have everything in place to prove the convergence of the Riemannian steepest descent (RSD) method on the Grassmann manifold for minimizing *f*. Starting from a subspace $\mathcal {X}_0 \in {{\,\textrm{Gr}\,}}(n,k)$, we iterate

21 $$\begin{aligned} \mathcal {X}_{t+1}={{\,\textrm{Exp}\,}}_{\mathcal {X}_t} (-\eta _t \, {{\,\textrm{grad}\,}}f(\mathcal {X}_t) ). \end{aligned}$$

Here, $\eta _t > 0$ is a step size that may depend on the iteration *t* and will be carefully chosen depending on the specific case, but always depending on $\gamma$, which equals $2 (\lambda _1-\lambda _n)$.

We start by a general result which shows that the distance to the optimal subspace contracts after one step of steepest descent. The step size depends on the smoothness and weak-quasi-convexity constants of *f* from Propositions 1 and 3. This is crucial since the constant $a(\mathcal {X})$ depends on the biggest principal angle between $\mathcal {X}$ and $\mathcal {V}_{\alpha }$ and bounding the evolution of distances of the iterates to the minimizer will help us also bound this constant.^5^ An alternative contraction property with a more tractable step size is presented in Proposition 6 of Appendix B.

### Lemma 5.1

(Contraction of RSD) Let $\mathcal {X}_t$ and $\mathcal {V}_\alpha$ have principal angles $0 \le \theta _1 \le \cdots \le \theta _k < \pi /2$. Then, iteration (21) with $0 \le \eta _t \le a(\mathcal {X}_t)/\gamma$ satisfies

$$\begin{aligned} {{\,\textrm{dist}\,}}^2(\mathcal {X}_{t+1},\mathcal {V}_{\alpha }) \le \big (1- 2 c_Q \delta a(\mathcal {X}_t) \, \eta _t \big ) {{\,\textrm{dist}\,}}^2(\mathcal {X}_t,\mathcal {V}_{\alpha }). \end{aligned}$$

Observe that $\gamma = 0$ implies $A = \lambda _1 I$ and any subspace $\mathcal {X}$ of dimension *k* will be an eigenspace of *A* with ${{\,\textrm{dist}\,}}(\mathcal {X},\mathcal {V}_\alpha )=0$. We will therefore not explicitly prove this lemma and all forthcoming convergence results for $\gamma =0$ since the statements will be trivially true.

### Proof of Lemma 5.1

By the assumption on the principal angles, we get that $0< a(\mathcal {X}_t) = \theta _k / \tan \theta _k \le 1$. The hypothesis on $\eta _t$ and Lemma 4.1 then gives

$$\begin{aligned} \eta _t \Vert {{\,\textrm{grad}\,}}f(\mathcal {X}_t)\Vert _2 \le \frac{a(\mathcal {X}_t)}{\gamma } \Vert {{\,\textrm{grad}\,}}f(\mathcal {X}_t)\Vert _2 \le \frac{1}{2} < \frac{\pi }{2}. \end{aligned}$$

By (5), this guarantees that the geodesic $\tau \mapsto {{\,\textrm{Exp}\,}}(- \tau \eta _t \, {{\,\textrm{grad}\,}}f(\mathcal {X}_t))$ lies within the injectivity domain at ${\mathcal {X}_t}$ for $\tau \in [0,1]$. Hence, ${{\,\textrm{Exp}\,}}$ is bijective along this geodesic and thus ${{\,\textrm{Log}\,}}_{\mathcal {X}_t}(\mathcal {X}_{t+1}) = -\eta _t \, {{\,\textrm{grad}\,}}f(\mathcal {X}_t)$. We can thus apply Lemma 3.1 to obtain

22 $$\begin{aligned} {{\,\textrm{dist}\,}}^2(\mathcal {X}_{t+1},\mathcal {V}_{\alpha })&\le \Vert -\eta _t {{\,\textrm{grad}\,}}f(\mathcal {X}_t)-\text {Log}_{\mathcal {X}_t}(\mathcal {V}_{\alpha }) \Vert ^2 \nonumber \\&= \eta _t^2 \Vert {{\,\textrm{grad}\,}}f(\mathcal {X}_t)\Vert ^2 + {{\,\textrm{dist}\,}}^2(\mathcal {X}_t, \mathcal {V}_{\alpha }) +2 \eta _t \, \sigma \ \end{aligned}$$

with

$$\begin{aligned} \sigma := \langle {{\,\textrm{grad}\,}}f(\mathcal {X}_t),{{\,\textrm{Log}\,}}_{\mathcal {X}_t}(\mathcal {V}_{\alpha }) \rangle . \end{aligned}$$

Theorem 4.1 and Lemma 4.2 together with Proposition 1 give

$$\begin{aligned} \frac{\sigma }{a(\mathcal {X}_t)}&\le f^*-f(\mathcal {X}_t)-c_Q \delta {{\,\textrm{dist}\,}}^2(\mathcal {X}_t,\mathcal {V}_{\alpha }) \\&\le -\frac{1}{2 \gamma } \Vert {{\,\textrm{grad}\,}}f(\mathcal {X}_t) \Vert ^2- c_Q \delta {{\,\textrm{dist}\,}}^2(\mathcal {X}_t,\mathcal {V}_{\alpha }). \end{aligned}$$

Multiplying by $2 a(\mathcal {X}_t)\, \eta _t$ and using $\eta _t \le a(\mathcal {X}_t) / \gamma$, we get

$$\begin{aligned} 2 \eta _t \, \sigma&\le -\frac{a(\mathcal {X}_t) \, \eta _t }{\gamma } \Vert {{\,\textrm{grad}\,}}f(\mathcal {X}_t) \Vert ^2 - 2 c_Q \delta a(\mathcal {X}_t) \, \eta _t \, {{\,\textrm{dist}\,}}^2(\mathcal {X}_t,\mathcal {V}_{\alpha }) \\  &\le -\eta _t^2 \Vert {{\,\textrm{grad}\,}}f(\mathcal {X}_t) \Vert ^2 - 2 c_Q \delta a(\mathcal {X}_t)\, \eta _t \, {{\,\textrm{dist}\,}}^2(\mathcal {X}_t, \mathcal {V}_{\alpha }). \end{aligned}$$

Substituting into (22), we obtain the first statement of the lemma. $\square$

### Remark 5.1

When $\delta =0$, Lemma 5.1 still holds *for any subspace* $\mathcal {V}_{\alpha }$ *spanned by* *k* *leading eigenvectors of* *A*. In that case, the lemma only guarantees that the distance between the iterates of steepest descent and this $\mathcal {V}_{\alpha }$ does not increase.

dummy

### Linear Convergence Rate Under Positive Eigengap

Lemma 5.1 features a contraction rate only for one step of the algorithm. In order to get a global convergence rate, one needs to bound the quantity $a(\mathcal {X}_t)$ from below and independently of *t*. To that end, we need a stricter bound in the distance of the initial guess to the optimum. Such a bound guarantees that $a(X_t)$ remains always lower bounded by a positive number, or equivalently, that the iterates of the algorithm never get too close to a non-optimal critical point.

#### Theorem 5.1

If ${{\,\textrm{dist}\,}}(\mathcal {X}_0,\mathcal {V}_{\alpha }) < \pi / 2$ then the iterates $\mathcal {X}_t$ of Riemannian steepest descent (21) with step size $\eta _t$ such that

$$\begin{aligned} 0<\eta \le \eta _t \le \cos ({{\,\textrm{dist}\,}}(\mathcal {X}_0,\mathcal {V}_{\alpha })) / \gamma \end{aligned}$$

satisfy

$$\begin{aligned} \text {dist}^2(\mathcal {X}_t,\mathcal {V}_{\alpha }) \le \left( 1- 2 c_Q \cos ({{\,\textrm{dist}\,}}(\mathcal {X}_0,\mathcal {V}_{\alpha }))\, \delta \, \eta \right) ^ t {{\,\textrm{dist}\,}}^2(\mathcal {X}_0,\mathcal {V}_{\alpha }). \end{aligned}$$

#### Proof

We first claim that ${{\,\textrm{dist}\,}}(\mathcal {X}_{t},\mathcal {V}_{\alpha }) \le {{\,\textrm{dist}\,}}(\mathcal {X}_0,\mathcal {V}_{\alpha })$ for all $t \ge 0$. This would then also imply that $\theta _k(\mathcal {X}_t, \mathcal {V}_\alpha ) < \pi /2$ for all $t\ge 0$ since

$$\begin{aligned} \theta _k(\mathcal {X}_t, \mathcal {V}_\alpha ) \le \sqrt{\sum _{i=1}^k \theta _i (\mathcal {X}_t, \mathcal {V}_\alpha )^2} = {{\,\textrm{dist}\,}}(\mathcal {X}_t, \mathcal {V}_\alpha ). \end{aligned}$$

For $t=0$, we have $\theta _k(\mathcal {X}_{0},\mathcal {V}_{\alpha }) < \pi /2$ by hypothesis on $\mathcal {X}_0$ and thus

$$\begin{aligned} a(\mathcal {X}_0)=\frac{\theta _k(\mathcal {X}_0,\mathcal {V}_{\alpha })}{\tan (\theta _k(\mathcal {X}_0,\mathcal {V}_{\alpha }))} \ge \cos (\theta _k(\mathcal {X}_0,\mathcal {V}_{\alpha })) \ge \cos ({{\,\textrm{dist}\,}}(\mathcal {X}_0,\mathcal {V}_{\alpha })). \end{aligned}$$

Since by construction $\eta _0 \le \cos ({{\,\textrm{dist}\,}}(\mathcal {X}_0,\mathcal {V}_{\alpha })) / \gamma$, this implies that $\eta _0 \le a(\mathcal {X}_0) / \gamma$ and Lemma 5.1 guarantees that ${{\,\textrm{dist}\,}}(\mathcal {X}_{1},\mathcal {V}_{\alpha }) \le \text {dist}(\mathcal {X}_0,\mathcal {V}_{\alpha })$. In particular, we also have $\theta _k(\mathcal {X}_{1},\mathcal {V}_{\alpha }) < \pi /2$.

Next, assume that

$$\begin{aligned} {{\,\textrm{dist}\,}}(\mathcal {X}_t,\mathcal {V}_{\alpha }) \le {{\,\textrm{dist}\,}}(\mathcal {X}_{0},\mathcal {V}_{\alpha }), \end{aligned}$$

which implies $\theta _k(\mathcal {X}_{t},\mathcal {V}_{\alpha }) < \pi /2$. Then by a similar argument like above, we have

23 $$\begin{aligned} a(\mathcal {X}_t) \ge \cos ({{\,\textrm{dist}\,}}(\mathcal {X}_t,\mathcal {V}_{\alpha })) \ge \cos ({{\,\textrm{dist}\,}}(\mathcal {X}_{0},\mathcal {V}_{\alpha })). \end{aligned}$$

By hypothesis on $\eta _t$, we observe

$$\begin{aligned} \eta _t \le \frac{\cos ({{\,\textrm{dist}\,}}(\mathcal {X}_0,\mathcal {V}_{\alpha }))}{\gamma } \le \frac{\cos ({{\,\textrm{dist}\,}}(\mathcal {X}_t,\mathcal {V}_{\alpha }))}{\gamma } \le \frac{a(\mathcal {X}_t)}{\gamma }. \end{aligned}$$

Applying Lemma 5.1 once again with the induction hypothesis proves the claim:

$$\begin{aligned} {{\,\textrm{dist}\,}}(\mathcal {X}_{t+1},\mathcal {V}_{\alpha }) \le {{\,\textrm{dist}\,}}(\mathcal {X}_t,\mathcal {V}_{\alpha }) \le {{\,\textrm{dist}\,}}(\mathcal {X}_0,\mathcal {V}_{\alpha }). \end{aligned}$$

The main statement of the theorem now follows easily: Since $\eta _t \le a(\mathcal {X}_t) / \gamma$ and $\theta _k(\mathcal {X}_{t}, \mathcal {V}_{\alpha }) < \pi /2$ for all $t\ge 0$, Lemma 5.1 gives

$$\begin{aligned} \text {dist}^2(\mathcal {X}_{t+1},\mathcal {V}_{\alpha }) \le \left( 1- 2 c_Q a(\mathcal {X}_t) \delta \eta _t \right) \text {dist}^2(\mathcal {X}_t,\mathcal {V}_{\alpha }). \end{aligned}$$

Combining with (23) and $\eta _t \ge \eta$ shows the desired result by induction. $\square$

If the eigengap $\delta$ is strictly positive, then Theorem 5.1 gives an exponential convergence rate towards the optimum $\mathcal {V}_{\alpha }$. If $\delta =0$, then Theorem 5.1*does not provide a convergence rate* but rather implies that the intrinsic distances of the iterates to the optimum do not increase.

From Theorem 5.1 we get immediately the following iteration complexity.

#### Corollary 5.1

Let Riemannian steepest descent be started from a subspace $\mathcal {X}_0$ that satisfies ${{\,\textrm{dist}\,}}(\mathcal {X}_0,\mathcal {V}_{\alpha }) < \pi /2$ and with step-size $\eta$ satisfying the condition of Theorem 5.1. Then after at most

$$\begin{aligned} T = 2 \frac{\log (\varepsilon ) - \log ({{\,\textrm{dist}\,}}(\mathcal {X}_0,\mathcal {V}_{\alpha }))}{\log (1- 0.8 \cos ({{\,\textrm{dist}\,}}(\mathcal {X}_0,\mathcal {V}_{\alpha })) \delta \eta )} +1 \le {\mathcal {O}}\left( \frac{\log ({{\,\textrm{dist}\,}}(\mathcal {X}_0,\mathcal {V}_{\alpha })) - \log (\varepsilon )}{\cos ({{\,\textrm{dist}\,}}(\mathcal {X}_0,\mathcal {V}_{\alpha })) \delta \eta } \right) \end{aligned}$$

many iterations, $\mathcal {X}_T$ will satisfy ${{\,\textrm{dist}\,}}(\mathcal {X}_T,\mathcal {V}_{\alpha }) \le \varepsilon$. With the maximal step size allowed in Theorem 5.1, we get

$$\begin{aligned} T \le {\mathcal {O}}\left( \frac{\lambda _1 - \lambda _n}{\delta } \frac{1}{\cos ^2({{\,\textrm{dist}\,}}(\mathcal {X}_0,\mathcal {V}_{\alpha }))} \log \left( \frac{{{\,\textrm{dist}\,}}(\mathcal {X}_0,\mathcal {V}_{\alpha })}{\varepsilon }\right) \right) . \end{aligned}$$

#### Proof

In order to guarantee $\text {dist}(\mathcal {X}_T,\mathcal {V}_{\alpha }) \le \epsilon$, it suffices to have

$$\begin{aligned} \left( 1- 2 c_Q \cos ({{\,\textrm{dist}\,}}(\mathcal {X}_0,\mathcal {V}_{\alpha }))\, \delta \, \eta \right) ^ T {{\,\textrm{dist}\,}}^2(\mathcal {X}_0,\mathcal {V}_{\alpha }) \le \epsilon ^2. \end{aligned}$$

Taking the logarithm of both sides, we get

$$\begin{aligned} T \log (1- 2 c_Q \cos ({{\,\textrm{dist}\,}}(\mathcal {X}_0,\mathcal {V}_{\alpha })) \delta \eta ) + 2 \log ({{\,\textrm{dist}\,}}(\mathcal {X}_0,\mathcal {V}_{\alpha })) \le 2 \log (\epsilon ), \end{aligned}$$

which gives

$$\begin{aligned} T \ge 2 \frac{ \log (\epsilon ) - \log ({{\,\textrm{dist}\,}}(\mathcal {X}_0,\mathcal {V}_{\alpha }))}{\log (1- 2 c_Q \cos ({{\,\textrm{dist}\,}}(\mathcal {X}_0,\mathcal {V}_{\alpha }))}, \end{aligned}$$

since $\log (1- 2 c_Q \cos ({{\,\textrm{dist}\,}}(\mathcal {X}_0,\mathcal {V}_{\alpha }))$ is negative. By considering that $c_Q \ge 0.8$, we get

$$\begin{aligned} T \ge 2 \frac{ \log (\epsilon ) - \log ({{\,\textrm{dist}\,}}(\mathcal {X}_0,\mathcal {V}_{\alpha }))}{\log (1- 0.8 \cos ({{\,\textrm{dist}\,}}(\mathcal {X}_0,\mathcal {V}_{\alpha })) \delta \eta )}, \end{aligned}$$

and the smallest integer that satisfies this inequality is exactly

$$\begin{aligned} T = 2 \frac{ \log (\epsilon ) - \log ({{\,\textrm{dist}\,}}(\mathcal {X}_0,\mathcal {V}_{\alpha }))}{\log (1- 0.8 \cos ({{\,\textrm{dist}\,}}(\mathcal {X}_0,\mathcal {V}_{\alpha })) \delta \eta )}. \end{aligned}$$

The inequality part of the result follows by considering that

$$\begin{aligned} \log (1- 0.8 \cos ({{\,\textrm{dist}\,}}(\mathcal {X}_0,\mathcal {V}_{\alpha })\delta \eta ) \ge - \cos ({{\,\textrm{dist}\,}}(\mathcal {X}_0,\mathcal {V}_{\alpha })) \delta \eta . \end{aligned}$$

The final bound for *T* follows by a simple substitution of $\eta =\cos ({{\,\textrm{dist}\,}}(\mathcal {X}_0,\mathcal {V}_{\alpha })) / \gamma$.

As expected, *T* depends inversely proportional on the eigengap $\delta$ and proportional to the spread of the eigenvalues. In addition, we also have an extra term $1/\cos ^2({{\,\textrm{dist}\,}}(\mathcal {X}_0,\mathcal {V}_{\alpha }))$ that depends on the initial distance ${{\,\textrm{dist}\,}}(\mathcal {X}_0,\mathcal {V}_{\alpha })$, which is due to the weak-quasi-convexity property of *f*. This is a conservative overestimation, since this quantity improves as the iterates get closer to the optimum.

#### Remark 5.2

If $\delta >0$, the exponential convergence rate is in terms of the intrinsic distance on the Grassmann manifold, that is, the $\ell _2$ norm of the principal angles. Standard convergence results for subspace iteration are stated for the biggest principal angle, that is, the $\ell _\infty$ norm. This is weaker than the intrinsic distance. For subspace iteration with projection, the convergence result from [31, Thm. 5.2] shows that all principal angles $\theta _i$ converge to zero and eventually gives convergence of the $\ell _4$ norm of the principal angles. This is also weaker than the intrinsic distance.

### Convergence of Function Values Without an Eigengap Assumption

When $\delta =0$, Theorem 5.1 still holds, but does not provide a rate of convergence as discussed above. Instead, we can prove the following result:

#### Theorem 5.2

If the distance ${{\,\textrm{dist}\,}}(\mathcal {X}_0,\mathcal {V}_{\alpha })$ of the initial subspace $\mathcal {X}_0$ to the minimizer satisfies ${{\,\textrm{dist}\,}}(\mathcal {X}_0,\mathcal {V}_{\alpha })<\pi / 2$ for a subspace $\mathcal {V}_{\alpha }$ that is spanned by any *k* leading eigenvectors of *A*, then the iterates $\mathcal {X}_t$ of Riemannian steepest descent (21) with fixed step size

$$\begin{aligned} \eta \le \cos ({{\,\textrm{dist}\,}}(\mathcal {X}_0,\mathcal {V}_{\alpha })) / \gamma \end{aligned}$$

satisfy

$$\begin{aligned} f(\mathcal {X}_t) - f^* \le \frac{2\gamma +\frac{1}{\eta }}{4 (\cos ({{\,\textrm{dist}\,}}(\mathcal {X}_0,\mathcal {V}_{\alpha }))t+1)} {{\,\textrm{dist}\,}}^2(\mathcal {X}_0,\mathcal {V}_{\alpha })={\mathcal {O}}\left( \frac{1}{t} \right) . \end{aligned}$$

#### Proof

Since we satisfy all the hypotheses of Theorem 5.1, we know that for all $t\ge 0$ it holds $\text {dist}(\mathcal {X}_{t},\mathcal {V}_{\alpha }) \le \text {dist}(\mathcal {X}_0,\mathcal {V}_{\alpha }) < \pi /2$ and thus also that $\mathcal {X}_t$ is in the injectivity domain of ${{\,\textrm{Exp}\,}}$ at $\mathcal {V}_{\alpha }$. In addition, its proof states in (23) that

$$\begin{aligned} a(\mathcal {X}_t) \ge C_0:= \cos ({{\,\textrm{dist}\,}}(\mathcal {X}_{0},\mathcal {V}_{\alpha })) > 0, \end{aligned}$$

which implies that the function *f* is weakly-quasi-convex at every $\mathcal {X}_t$ with constant $2 C_0$. Hence

24 $$\begin{aligned} 2 C_0 \Delta _t \le \langle {{\,\textrm{grad}\,}}f(\mathcal {X}_t), - {{\,\textrm{Log}\,}}_{\mathcal {X}_t} (\mathcal {V}_{\alpha }) \rangle , \end{aligned}$$

where we defined

$$\begin{aligned} \Delta _t:= f(\mathcal {X}_t) - f^*. \end{aligned}$$

Similar to the proof of Theorem 5.1, by the hypothesis on the step size $\eta _t$, Lemma 5.1 shows that $\mathcal {X}_{t+1}$ is in the injectivity domain of ${{\,\textrm{Exp}\,}}$ at $\mathcal {X}_t$. Hence, by the definition of Riemannian steepest descent, we have

25 $$\begin{aligned} {{\,\textrm{Log}\,}}_{\mathcal {X}_t} (\mathcal {X}_{t+1})=-\eta {{\,\textrm{grad}\,}}f(\mathcal {X}_t). \end{aligned}$$

In addition, the smoothness property (10) of *f* gives

$$\begin{aligned} \Delta _{t+1}-\Delta _t \le \langle {{\,\textrm{grad}\,}}f(\mathcal {X}_t), {{\,\textrm{Log}\,}}_{\mathcal {X}_t} (\mathcal {X}_{t+1}) \rangle +\frac{\gamma }{2} {{\,\textrm{dist}\,}}^2(\mathcal {X}_t,\mathcal {X}_{t+1}). \end{aligned}$$

Substituting (25), we obtain

26 $$\begin{aligned} \Delta _{t+1}-\Delta _t \le \left( -\eta +\frac{\gamma }{2} \eta ^2 \right) \Vert {{\,\textrm{grad}\,}}f(\mathcal {X}_t)\Vert ^2 \le 0, \end{aligned}$$

since $\eta \le C_0/\gamma$ with $0 < C_0:= \cos ({{\,\textrm{dist}\,}}(\mathcal {X}_0, \mathcal {V}_{\alpha })) \le 1$ and $\gamma > 0$.

Since ${{\,\textrm{Gr}\,}}(n,k)$ has nonnegative sectional curvature, Lemma 3.2 implies

$$\begin{aligned} {{\,\textrm{dist}\,}}^2(\mathcal {X}_{t+1}, \mathcal {V}_{\alpha }) \le {{\,\textrm{dist}\,}}^2(\mathcal {X}_t, \mathcal {X}_{t+1})+ {{\,\textrm{dist}\,}}^2(\mathcal {X}_t, \mathcal {V}_{\alpha })-2 \langle {{\,\textrm{Log}\,}}_{\mathcal {X}_t} (\mathcal {X}_{t+1}), {{\,\textrm{Log}\,}}_{\mathcal {X}_t} (\mathcal {V}_{\alpha }) \rangle . \end{aligned}$$

Substituting (25) into the above and rearranging terms gives

$$\begin{aligned} 2 \eta \langle {{\,\textrm{grad}\,}}f(\mathcal {X}_t), - {{\,\textrm{Log}\,}}_{\mathcal {X}_t} (\mathcal {V}_{\alpha }) \rangle \le {{\,\textrm{dist}\,}}^2(\mathcal {X}_t, \mathcal {V}_{\alpha })-{{\,\textrm{dist}\,}}^2(\mathcal {X}_{t+1}, \mathcal {V}_{\alpha })+ \eta ^2 \Vert {{\,\textrm{grad}\,}}f(\mathcal {X}_t) \Vert ^2. \end{aligned}$$

Combining with (24), we get

27 $$\begin{aligned} \Delta _t \le \frac{1}{4 C_0 \eta } ( {{\,\textrm{dist}\,}}^2(\mathcal {X}_t, \mathcal {V}_{\alpha })-{{\,\textrm{dist}\,}}^2(\mathcal {X}_{t+1}, \mathcal {V}_{\alpha })) + \frac{\eta }{4 C_0} \Vert {{\,\textrm{grad}\,}}f(\mathcal {X}_t) \Vert ^2. \end{aligned}$$

Now multiplying (26) by $\frac{1}{C_0}$ and summing with (27) gives

28 $$\begin{aligned}  &   \frac{1}{C_0} \Delta _{t+1} - \left( \frac{1}{ C_0} - 1 \right) \Delta _t \le \frac{1}{4 C_0 \eta } ( {{\,\textrm{dist}\,}}^2(\mathcal {X}_t, \mathcal {V}_{\alpha })-{{\,\textrm{dist}\,}}^2(\mathcal {X}_{t+1}, \mathcal {V}_{\alpha })) \nonumber \\  &   \quad +\frac{1}{C_0} \left( -\eta + \frac{\gamma }{2}\eta ^ 2 + \frac{\eta }{4} \right) \Vert {{\,\textrm{grad}\,}}f(\mathcal {X}_t) \Vert ^2. \end{aligned}$$

By assumption $\eta \le C_0/\gamma$, where $0 < C_0:= \cos ({{\,\textrm{dist}\,}}(\mathcal {X}_0, \mathcal {V}_{\alpha })) \le 1$ and $\gamma > 0$. Since

$$\begin{aligned} \frac{\eta }{C_0} \left( -1 + \frac{\gamma }{2} \eta + \frac{1}{4} \right) \le \frac{\eta }{C_0} \left( \frac{C_0}{2} -\frac{3}{4} \right) \le - \frac{1}{4} \frac{\eta }{C_0}< 0. \end{aligned}$$

Inequality (28) can be simplified to

$$\begin{aligned} \frac{1}{C_0} \Delta _{t+1} - \left( \frac{1}{ C_0} - 1 \right) \Delta _t \le \frac{1}{4 C_0 \eta } ( {{\,\textrm{dist}\,}}^2(\mathcal {X}_t, \mathcal {V}_{\alpha })-{{\,\textrm{dist}\,}}^2(\mathcal {X}_{t+1}, \mathcal {V}_{\alpha })). \end{aligned}$$

Summing from 0 to $t-1$ gives

$$\begin{aligned} \frac{1}{C_0} \Delta _t + \sum _{s=1}^{t-1} \Delta _s - \left( \frac{1}{C_0} -1 \right) \Delta _0 \le \frac{1}{4 C_0 \eta } \left( {{\,\textrm{dist}\,}}^2(\mathcal {X}_0, \mathcal {V}_{\alpha }) - {{\,\textrm{dist}\,}}^2(\mathcal {X}_t, \mathcal {V}_{\alpha }) \right) . \end{aligned}$$

From the smoothness property (10) at the critical point $\mathcal {V}_\alpha$ of *f*, we get

$$\begin{aligned} \Delta _0 \le \frac{\gamma }{2} {{\,\textrm{dist}\,}}^2(\mathcal {X}_0, \mathcal {V}_{\alpha }). \end{aligned}$$

Combining these two inequalities then leads to

$$\begin{aligned} \frac{1}{C_0} \Delta _t + \sum _{s=0}^{t-1} \Delta _s&\le \frac{1}{C_0} \Delta _0 + \frac{1}{4 C_0 \eta } {{\,\textrm{dist}\,}}^2(\mathcal {X}_0, \mathcal {V}_{\alpha }) \\&\le \frac{1}{2C_0} \left( \gamma +\frac{1}{2 \eta }\right) {{\,\textrm{dist}\,}}^2(\mathcal {X}_0, \mathcal {V}_{\alpha }). \end{aligned}$$

Since (26) holds for all $t \ge 0$, it also implies $\Delta _t \le \Delta _s$ for all $1 \le s \le t$. Substituting

$$\begin{aligned} t \Delta _t \le \sum _{s=0}^{t-1} \Delta _s \end{aligned}$$

into the inequality from above,

$$\begin{aligned} \Delta _t \le \frac{1}{2C_0} \frac{\gamma +\frac{1}{2 \eta }}{\frac{1}{C_0}+t} {{\,\textrm{dist}\,}}^2(\mathcal {X}_0, \mathcal {V}_{\alpha }) = \frac{\gamma +\frac{1}{2\eta }}{2(C_0 t+1)} {{\,\textrm{dist}\,}}^2(\mathcal {X}_0, \mathcal {V}_{\alpha }), \end{aligned}$$

we obtain the desired result. $\square$

#### Remark 5.3

This type of result is standard for functions that are geodesically convex (see, e.g. [35]). Our objective function does not satisfy this property, but we can still have a similar upper bound on the iteration complexity for convergence in function value. We note that this does not imply convergence of the iterates to a specific *k*-dimensional subspace, but only convergence of a subsequence of the sequence of the iterates.

### Sufficiently Small Step Sizes

The convergence results in Theorems 5.1 and 5.2 require that the initial subspace $\mathcal {X}_0$ lies within a distance strictly less than $\pi /2$ from a global minimizer $\mathcal {V}_{\alpha }$. While this condition is independent from the eigengap (unlike results that rely on standard convexity, see appendix), it is also not fully satisfactory: it is hard to verify in practice, and it is unnecessarily severe in numerical experiments. In fact, this condition is only used to obtain a uniform lower bound on the weak-quasi-convexity constant $a(\mathcal {X}_t) = \theta _k^{(t)} / \tan (\theta _k^{(t)})$ with $\theta _k^{(t)}$ the largest principal angle between $\mathcal {X}_t$ and $\mathcal {V}_{\alpha }$. Since the Riemannian distance is the $\ell _2$ norm of the principal angles, a contraction in this distance leads automatically to $\theta _k^{(t)} < \pi /2$ if $\theta _k^{(0)} < \pi /2$. If one could guarantee by some other reasoning that $\theta _k^{(t)}$ does not increase after one step, the condition ${{\,\textrm{dist}\,}}(\mathcal {X}_0, \mathcal {V}_\alpha ) < \pi /2$ would not be needed.

We now show that for sufficiently small step sizes $\eta _t$, the largest principal angle $\theta _k^{(t)}$ between $\mathcal {X}_t$ and $\mathcal {V}_{\alpha }$ does indeed not increase after each iteration of Riemannian steepest descent regardless of the initial subspace $\mathcal {X}_0$. While it does not explain what we observe in numerical experiments where large steps can be taken, it is a first result in explaining why we can initialize the iteration at a random initial subspace $\mathcal {X}_0$.

#### Proposition 5

Riemannian steepest descent started from a subspace $\mathcal {X}_t$ returns a subspace $\mathcal {X}_{t+1}$ such that

$$\begin{aligned} \theta _k(\mathcal {X}_{t+1},\mathcal {V}_{\alpha }) \le \theta _k(\mathcal {X}_t,\mathcal {V}_{\alpha }), \end{aligned}$$

for all step sizes $0 \le \eta \le \bar{\eta }$ where $\bar{\eta }> 0$ is sufficiently small.

For the proof of this proposition, we will need the derivatives of certain singular values. While this is well known for isolated singular values, it is possible to generalize to higher multiplicities as well by relaxing the ordering and sign of singular values [10]. For a concrete formula, we use the following result from Lemma A.5 in [24].

#### Lemma 5.2

Let $\sigma _1 \ge \cdots \ge \sigma _n$ be the singular values of *S* $\in \mathbb {R}^{n \times n}$ with $u_{1}, \ldots , u_{n}$ and $v_{1}, \ldots , v_{n}$ the associated left and right orthonormal singular vectors. Suppose that $\sigma _j$ has multiplicity *m*, that is,

$$ \sigma _{j_0-1}> \sigma _{j_0} = \cdots = \sigma _j =\cdots = \sigma _{j_0+m-1} > \sigma _{j_0+m}. $$

Then, the *j*th singular value of $S+\eta$T satisfies

$$\begin{aligned} \sigma _j(S+\eta T) = \sigma _j + \eta \lambda _{j - j_0 + 1} + {\mathcal {O}}(\eta ^2), \quad \eta \rightarrow 0^+, \end{aligned}$$

where $\lambda _{j}$ is the *j*th largest eigenvalue of $\tfrac{1}{2}(U^T B V + V^T B^T U)$ with

$$\begin{aligned} U = \begin{bmatrix} u_{j_0}&\cdots&u_{j_0+m-1} \end{bmatrix} \quad \text {and}\quad V = \begin{bmatrix} v_{j_0}&\cdots&v_{j_0+m-1} \end{bmatrix}. \end{aligned}$$

#### Proof of Proposition 5

For ease of notation, let $X:= X_t$ and $X_+:= X_{t+1}$ such that $\mathcal {X}_t = {{\,\textrm{span}\,}}(X)$ and $\mathcal {X}_{t+1} = {{\,\textrm{span}\,}}(X_+)$. By definition of the exponential map on Grassmann, the next iterate of the Riemannian SD iteration (21) with step $\eta$ satisfies

$$\begin{aligned} X_+=X V \cos (\eta \Sigma ) V^T + U \sin (\eta \Sigma ) V^T \end{aligned}$$

where

$$\begin{aligned} U \Sigma V^T = -{{\,\textrm{grad}\,}}f(\mathcal {X}_t). \end{aligned}$$

Since *V* is orthogonal, we can write

$$\begin{aligned} U \sin (\eta \Sigma ) V^T = U (\eta \Sigma ) V^T V \left( \frac{\sin (\eta \Sigma )}{\eta \Sigma } \right) V^T = -\eta {{\,\textrm{grad}\,}}f(\mathcal {X}_t) V \left( \frac{\sin (\eta \Sigma )}{\eta \Sigma } \right) V^T \end{aligned}$$

where $1/\Sigma :=\Sigma ^{-1}$ and $\frac{\sin 0}{0} = 1$. Taking Taylor expansions of $\sin$ and $\cos$,

$$\begin{aligned} V \cos (\eta \Sigma ) V^T&= V \left( I - {\mathcal {O}}(\eta ^2) \right) V^T = I-{\mathcal {O}}(\eta ^2) \\ V \frac{\sin (\eta \Sigma )}{\eta \Sigma } V^T&= V \left( I - {\mathcal {O}}(\eta ^2) \right) V^T = I-{\mathcal {O}}(\eta ^2), \end{aligned}$$

we obtain

29 $$\begin{aligned} {V}_{\alpha }^T X_+&= {V}_{\alpha }^T X (I-{\mathcal {O}}(\eta ^2)) + {V}_{\alpha }^T (- \eta {{\,\textrm{grad}\,}}f(\mathcal {X})) (I-{\mathcal {O}}(\eta ^2)) \nonumber \\&= {V}_{\alpha }^T (X- \eta {{\,\textrm{grad}\,}}f(\mathcal {X}_t)) (I-{\mathcal {O}}(\eta ^2)) \end{aligned}$$

since $$\Vert V_\alpha \Vert _2 = \Vert X \Vert _2=1$$.

Let now $\theta$ be the vector of *k* principal angles between $\mathcal {X}_t$ and $\mathcal {V}_\alpha$. As in (12) and (19), we therefore have the SVDs

30 $$\begin{aligned} V_\alpha ^T X = U_1 \cos \theta \, V_1^T \qquad \text {and} \qquad V_\beta ^T X = \tilde{U}_2 \sin \theta \, V_1^T, \end{aligned}$$

where $U_1,V_1 \in \mathbb {R}^{k \times k}$ and $\tilde{U}_2 \in \mathbb {R}^{(n-k) \times k}$ have orthonormal columns. Next, we write (29) in terms of

$$\begin{aligned} M:=\sin ^2 \theta \, U_1^T \Lambda _{\alpha } U_1 \cos \theta - \cos \theta \sin \theta \, \tilde{U}_2^T \Lambda _{\beta } \tilde{U}_2 \sin \theta . \end{aligned}$$

Since ${{\,\textrm{grad}\,}}f(\mathcal {X}_t) = -2 (I-XX^T)AX$, the identity (17) gives

$$\begin{aligned} {V}_{\alpha }^T (X- \eta {{\,\textrm{grad}\,}}f(\mathcal {X}_t)) = V_\alpha ^T X +2 \eta \Lambda _\alpha V_\alpha ^T X - 2 \eta V_\alpha ^T X X^T A X. \end{aligned}$$

After substituting (11) and (30), a short calculation using $\cos ^2 \theta = I - \sin ^2 \theta$ and the orthogonality of $U_1$ and $V_1$ then shows

$$\begin{aligned} V_{\alpha }^T (X- \eta {{\,\textrm{grad}\,}}f(\mathcal {X}_t)) = U_1 (\cos \theta + 2\eta M) V_1 ^T. \end{aligned}$$

Relating back to (29), we thus obtain

$$\begin{aligned} V_{\alpha }^T {X}_{+}&= U_1 (\cos \theta + 2\eta M) V_1 ^T (I-{\mathcal {O}}(\eta ^2)) \\&= U_1 (\cos \theta + 2\eta M) (I - V_1 ^T {\mathcal {O}}(\eta ^2) V_1)V_1 ^T \\&= U_1 (\cos \theta + 2\eta M - {\mathcal {O}}(\eta ^2)) V_1^T. \end{aligned}$$

The singular values of $V_{\alpha }^T {X}_{+}$ are therefore the same as the singular values of the matrix $\cos \theta + 2\eta M +{\mathcal {O}}(\eta ^2)$.

By Weyl’s inequality (see, e.g., [18, Cor. 7.3.5]), each singular value of $\cos \theta + 2\eta M +{\mathcal {O}}(\eta ^2)$ is ${\mathcal {O}}(\eta ^2)$ close to some singular value of $\cos \theta + 2\eta M$. Let $1 \le j \le k$. Denote the *j*th singular value of $\cos \theta + 2\eta M$ by $\sigma _j(\eta )$ to which we will apply Lemma 5.2. Let *m* be the multiplicity of $\sigma _j(0)$. Hence, there exists $j_0$ such that $\sigma _{j_0}(0) = \cdots = \sigma _{j}(0) = \cdots = \sigma _{j_0+m-1}(0)$. Since $\cos \theta$ is a diagonal matrix with decreasing diagonal, its $\ell$th singular value equals $\cos \theta _\ell$ and its associated left/right singular vector is the $\ell$th canonical vector $e_\ell$. Denoting

$$\begin{aligned} E = \begin{bmatrix} e_{j_0}&\cdots&e_{j_0+m-1} \end{bmatrix}, \end{aligned}$$

observe that $\cos \theta \, E = \cos \theta _{j_0} \, E$ (here, $\cos \theta$ is a diagonal matrix and $\cos \theta _{j_0}$ is a scalar) and likewise for $\sin \theta \, E$. We thus get

$$\begin{aligned} E^T M E = \sin ^2 \theta _{j_0} \cos \theta _{j_0} (U_1^T \Lambda _{\alpha } U_1 - \tilde{U}_2^T \Lambda _{\beta } \tilde{U}_2 ). \end{aligned}$$

In the proof of Proposition 3, we showed that the matrix in brackets above is symmetric and positive semi-definite (see (20)). Since $0 \le \theta _{j_0} \le \pi /2$, the eigenvalues of $E^T M E$ are therefore all non-negative. Lemma 5.2 thus gives that $\sigma _j(\eta ) \ge \sigma _j$ for sufficiently small and positive $\eta$. Since the singular values of $V_\alpha ^T X_+$ are the cosines of the principal angles between $\mathcal {V}_\alpha$ and $\mathcal {X}_{t+1}$ with step size $\eta \ge 0$, we conclude that there exists $\bar{\eta }>0$ such that for all $\eta \in [0,\bar{\eta }]$ it holds

$$\begin{aligned} \theta _j(\mathcal {X}_{t+1}, \mathcal {V}_{\alpha }) \le \theta _j(\mathcal {X}_t, \mathcal {V}_{\alpha }). \end{aligned}$$

Since *j* was arbitrary, this finishes the proof. $\square$

## Numerical Experiment

We report on a small numerical experiment to verify the convergence rates proven above. The steepest descent iteration with fixed step size was implemented in Matlab using the geodesic formula (4).

As first test matrix, we took the standard 3D Laplacian on a unit cube, discretized with finite differences and zero Dirichlet boundary conditions. The size of the matrix *A* is $n=400$. We tested a few values for the block size *k*. They are depicted in the table below, together with other parameters that are relevant for Theorem 5.1.

**Table:** 

*k*	$\delta$	$\text {dist}\,(\mathcal {X}_0, \mathcal {V}_\alpha )$
1	$0.0665\ldots$	$0.113\ldots$
6	$0.0665\ldots$	$0.280\ldots$
10	$0.0262\ldots$	$0.350\ldots$

**Fig. 1:**
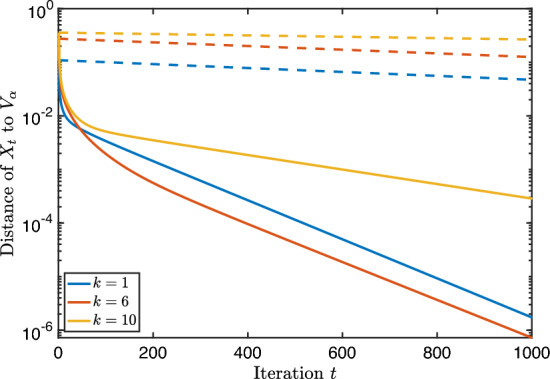
Steepest descent along geodesics for the block Rayleigh quotient of size *k* applied to a discretized 3D Laplacian matrix. The full lines correspond to the experimental values and the dashed lines to the theoretical upper bounds

In Fig. 1, the convergence of the Riemannian distance is visible in addition to the theoretical convergence rate of Theorem 5.1. We see that in all cases, these bounds on the convergence are valid (in particular, exponential) although they are rather conservative.

For completeness, we implemented steepest descent starting from a subspace $\mathcal {X}_0$ far away from the optimum. In that case, Theorem 5.1 does not apply since, if $\text {dist}(\mathcal {X}_0, \mathcal {V}_\alpha )>\frac{\pi }{2}$, the step-size $\eta \le \cos ({{\,\textrm{dist}\,}}(\mathcal {X}_0,\mathcal {V}_{\alpha })) / \gamma$ is or will become eventually negative. However, a meaningful choice for $\eta$ is given by Proposition 7 of Appendix B, where we prove a local linear convergence rate for the function values of the iterates for step size $\eta =1 / \gamma$.

**Fig. 2:**
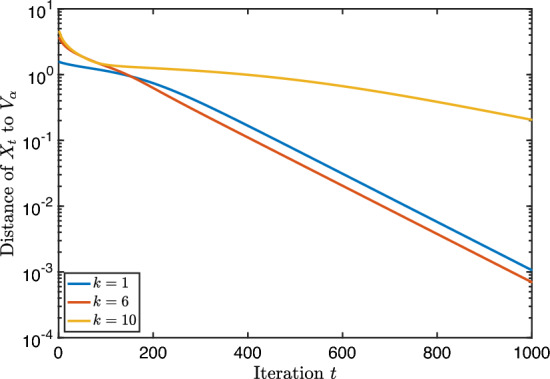
Same matrix from Fig. 1 but such that $\textrm{dist}(\mathcal {X}_0, \mathcal {V}_\alpha ) \gg \pi /2$ and with fixed step size $1/\gamma$

We see in Fig. 2 that despite the seemingly bad initial guess, steepest descent converges globally with a linear rate. This reveals that the restriction of the initial guess in our theoretical results is probably unnecessary and constitutes a topic for future work.

In the second test, we investigate the convergence when the eigengap $\delta$ is small or zero. In particular, we take $A=VDV^T \in \mathbb {R}^{1000 \times 1000}$ with *V* a random orthogonal matrix and *D* contains the eigenvalues

$$\begin{aligned} \lambda _1= 3,\ \lambda _2=2,\ \lambda _3 = 1+10^{-2}+10^{-6}, \ \lambda _4=1+10^{-6},\ \lambda _5=\lambda _6= 1. \end{aligned}$$

The other eigenvalues are equidistantly distributed between 0.1 and 0.2. The block size and other relevant parameters for the test are described below. Since the convergence for small $\delta$ slows down considerably after the first 5 iterations, we apply the bounds of Theorem 5.2 at iteration $t=6$ (and treat this as the start with $t=0$).

**Table:** 

*k*	$\delta$	$\text {dist}\,(\mathcal {X}_0, \mathcal {V}_\alpha )$	$\text {dist}\,(\mathcal {X}_6, \mathcal {V}_\alpha )$
2	$0.99\ldots$	$0.051\ldots$	$0.001\ldots$
3	$10^{-2}$	$0.055\ldots$	$0.031\ldots$
4	$10^{-6}$	$0.063\ldots$	$0.045\ldots$
5	0	$0.070\ldots$	$0.054\ldots$

**Fig. 3:**
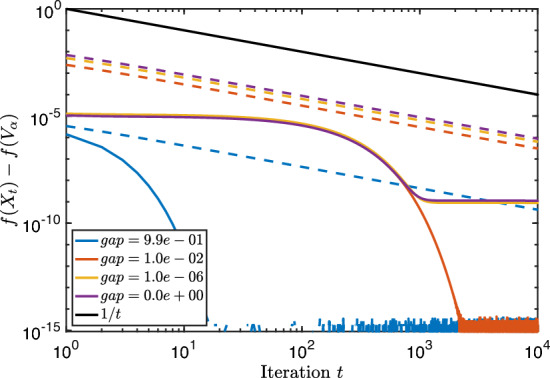
Steepest descent along geodesics for the block Rayleigh quotient of size *k* applied to a random matrix with small eigengaps. The full lines correspond to the experimental values and the dashed lines to the theoretical upper bounds of Theorem 5.2. Each color corresponds to a certain eigengap $\delta$

The convergence in function value is visible in Fig. 3. Observe that we have displayed a logarithmic scale for both axes whereas before the figure had a logarithmic scale only for *y*-axis. Algebraic convergence like 1/*t* is therefore visible as a straight line. We see in the figure that the convergence is not easily described, and that there is no clear difference between zero or small gap. However, the upper bounds of Theorem 5.2 are again valid. In addition, when the gap is not small, the convergence is clearly faster.

**Fig. 4:**
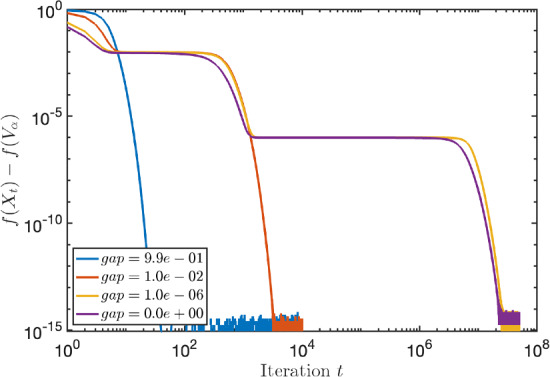
Same matrices with small eigengap from Fig. 3 but such that $\textrm{dist}(\mathcal {X}_0, \mathcal {V}_\alpha ) \gg \pi /2$ and with fixed step size $1/\gamma$

As before, we test the behaviour of steepest descent starting from an initial guess far away from the optimum. We use again step size $1/ \gamma$; see Theorem B.2. In Fig. 4 we show the convergence of steepest descent for the problem defined by matrix *A* with this step size.

We observe again that the local nature of our theoretical results is quite pessimistic: the algorithm converges with an algebraic rate even with a bad initial guess but it shows eventually linear convergence.

## Conclusion and Future Work

We provided the first non-asymptotic convergence rates for Riemannian steepest descent on the Grassmann manifold for computing a subspace spanned by *k* leading eigenvectors of a symmetric matrix *A*.

Our main idea was to exploit a convexity-like structure of the block Rayleigh quotient, which can be of much more general interest than for only analyzing steepest descent. One example is line search methods, which have usually favourable properties compared to vanilla steepest descent. Also, weakly-quasi-convex functions have been proven to admit accelerated algorithms [25], while accelerated or almost accelerated Riemannian algorithms have been developed in [5, 6, 36]. It would naturally be interesting to examine whether a provable accelerated method can be developed for the block Rayleigh quotient on the Grassmann manifold. This would hopefully reduce the dependence of the iteration complexity on the eigengap $\delta$ from ${\mathcal {O}}(1/\delta )$ to ${\mathcal {O}}(1/\sqrt{\delta })$.

Another interesting direction is to extend the analysis of [4] from the computation of just one leading eigenvector to computation of a whole subspace, using the generalized machinery developed in this work, or develop a noisy version of steepest descent and compare with noisy power method [14].

## Appendix A: Geodesic convexity

Let $\delta > 0$ and thus $\mathcal {V}_\alpha$ is the unique minimizer of *f*. Define the following neighbourhood of $\mathcal {V}_\alpha$ in ${{\,\textrm{Gr}\,}}(n,k)$:

31 $$\begin{aligned} N_*(\varphi ) = \{ \mathcal {X} \in {{\,\textrm{Gr}\,}}(n,k) :\theta _k(\mathcal {X}, \mathcal {V}_\alpha ) < \varphi \} \qquad \text {with}\,\varphi \in [0, \pi /4]. \end{aligned}$$

Here, $\theta _k(\mathcal {X}, \mathcal {V}_\alpha )$ denotes the largest principal angle between $\mathcal {X}$ and $\mathcal {V}_\alpha$. Since $\theta _k$ is a metric on ${{\,\textrm{Gr}\,}}(n,k)$ (see [30]), any two subspaces $\mathcal {X},\mathcal {Y} \in N_*(\varphi )$ will satisfy $\theta _k(\mathcal {X},\mathcal {Y}) < \pi /2$ by triangle inequality. They thus have a unique connecting geodesic. It is shown in [?, Lemma 2] that for any fixed $\varphi \in [0, \pi /4]$ this geodesic remains in $N_*(\varphi )$. Each set $N_*(\varphi )$ is thus an open totally geodesically convex set as defined in, e.g., [8, Def. 11.16].

One of the main results in [3], namely Cor. 4, states that *f* is geodesically convex on $N_*(\pi /4)$. This is unfortunately wrong and we present a small counterexample.

Counterexample for Cor. 4 in [3].

Here we use the notation of [3]. The reader is encouraged to take a look there for notational purposes.

Take $c:=\cos (\pi /4) = \sqrt{2}/2$ and $0 \le \varepsilon <1$. Define the matrices

$$ X_p:= \begin{pmatrix} 1 &  0 \\ 0 &  1 \\ 0 &  0 \\ 0 &  0 \end{pmatrix}, \quad U_p:= \begin{pmatrix} c &  0 \\ 0 &  c \\ c &  0 \\ 0 &  c \end{pmatrix}, \quad M:= U_p \begin{pmatrix} 1 &  0 \\ 0 &  \varepsilon \end{pmatrix}. $$

These matrices satisfy the conditions posed in [3]:

- Principal alignment: $$X_p^T U_p = \begin{pmatrix} c &  0 \\ 0 &  c \end{pmatrix}$$.
- Principal angles between $X_p$ and $U_p$ are in $[0,\pi /4]$.
- $$U = U_p$$ since $$Q=I$$.

Now consider the following tangent vector of unit Frobenius norm:

$$ \Delta = \begin{pmatrix} 0 &  0 \\ 0 &  0 \\ 0 &  1 \\ 0 &  0 \end{pmatrix}. $$

It is clearly a tangent vector of $[X_p]$ since $X_p^T \Delta = 0$. The Hessian of $f_{full}$ at $[X_p]$ in the direction of $\Delta$ satisfies (see equation (4.2) in [3])

$$ \text {Hess}f_{full}([X_p])[\Delta ,\Delta ] = -2 {{\,\textrm{Tr}\,}}(M^T \Delta \Delta ^T (I-X_p X_p^T) M) + \Vert (\Delta X_p^T + X_p\Delta ^T) M \Vert _F^2. $$

Simple calculation shows that

$$ \text {Hess}f_{full}([X_p])[\Delta ,\Delta ] = -2 c^2 + (1+\varepsilon ^2) c^2. $$

Hence for $\varepsilon <1$, we have $\text {Hess}f_{full}([X_p])[\Delta ,\Delta ]<0$ and the $f_{full}$ is non-convex which is in contrast with Corollary 4. $\square$

Instead, our Theorem A.1 guarantees convexity when $\varphi$ depends on the spectral gap. Since *f* is smooth, the function is geodesically convex on $N_*(\varphi )$ if and only if its Riemannian Hessian is positive definite on $N_*(\varphi )$; see, e.g., [8, Thm. 11.23]. We will therefore compute the eigenvalues of ${{\,\textrm{Hess}\,}}f$ based on its matrix representation. This requires us to first vectorize the tangent space.

From (3), a matrix *G* is a tangent vector if and only if $G^TX = 0$. Hence, taking $X_\perp \in \mathbb {R}^{n \times (n-k)}$ orthonormal such that $\mathcal {X}^\perp ={{\,\textrm{span}\,}}(X_\perp )$, we have the equivalent definition

$$\begin{aligned} T_X {{\,\textrm{Gr}\,}}(n,k) = \{ X_\perp M :M \in \mathbb {R}^{(n-k) \times k} \}. \end{aligned}$$

The matrix *M* above can be seen as the coordinates of $G=X_\perp M$ in the basis $X_\perp$. More specifically, by using the linear isomorphism ${{\,\textrm{vec}\,}}:\mathbb {R}^{n \times k} \rightarrow \mathbb {R}^{nk}$ that stacks all columns of a matrix under each other, we can define the tangent vectors of ${{\,\textrm{Gr}\,}}(n,k)$ as standard (column) vectors in the following way:

$$\begin{aligned} {{\,\textrm{vec}\,}}(G) = {{\,\textrm{vec}\,}}(X_\perp M ) = (I_k \otimes X_\perp ) {{\,\textrm{vec}\,}}(M). \end{aligned}$$

Here, the Kronecker product $\otimes$ appears due to [17, Lemma 4.3.1]. By well-known properties of $\otimes$ (see, e.g., [17, Chap. 4.2]), the matrix $I_k \otimes X_\perp$ has orthonormal columns. We have thus obtained an orthonormal basis for the (vectorized) tangent space. With this setup, we can now construct the Hessian.

### Lemma A.1

Let $I_k \otimes X_\perp$ be the orthonormal basis for the vectorization of $T_{\mathcal {X}} {{\,\textrm{Gr}\,}}(n,k)$. Then the Riemannian Hessian of *f* at $\mathcal {X}$ in that basis has the symmetric matrix representation

32 $$\begin{aligned} H_X = 2 (X^T A X \otimes I_{n-k} - I_k \otimes X_\perp ^T A X_\perp ). \end{aligned}$$

Furthermore, with $1 \le i \le k$ and $1 \le j \le n-k$ its $k(n-k)$ eigenvalues satisfy

$$\begin{aligned} \lambda _{i,j}(H_X) = 2(\lambda _i(X^T A X) - \lambda _j(X_\perp ^T A X_\perp )). \end{aligned}$$

### Proof

Since ${{\,\textrm{vec}\,}}$ is a linear isomorphism, the symmetric matrix $H_X$ satisfies

$$\begin{aligned} {{\,\textrm{Hess}\,}}f(X)[X_\perp M, X_\perp M] = \langle {{\,\textrm{vec}\,}}(M), H_X {{\,\textrm{vec}\,}}(M) \rangle , \qquad \forall M \in \mathbb {R}^{n \times (n-k)}, \end{aligned}$$

where $\langle \cdot , \cdot \rangle$ is the Euclidean inner product. Define $m = {{\,\textrm{vec}\,}}(M)$. Plugging in the formula (9) for ${{\,\textrm{Hess}\,}}f$, we calculate

$$\begin{aligned} {{\,\textrm{Hess}\,}}f(X)[X_\perp M, X_\perp M]&= 2\langle X_\perp M, X_\perp M X^T A X - AX_\perp M \rangle \\&= 2\langle (I \otimes X_\perp ) m, (X^T A X \otimes X_\perp ) m - (I \otimes AX_\perp ) m \rangle \\&= 2\langle m, (I \otimes X_\perp )^T (X^T A X \otimes X_\perp - I \otimes AX_\perp ) m \rangle \\&= 2\langle m, (X^T A X \otimes I - I \otimes X_\perp ^T AX_\perp ) m \rangle \end{aligned}$$

Here, we used typical calculus rules for the Kronecker product (see, e.g., [17, Chap. 4.2]). We recognize the matrix $H_X$ directly.

The eigenvalues of (32) can be directly obtained using [17, Thm. 4.4.5]. $\square$

Taking $X=V_\alpha$ and $X_\perp = V_\beta$, Lemma A.1 shows immediately that the minimal eigenvalue of ${{\,\textrm{Hess}\,}}f(\mathcal {V}_\alpha )$ is equal to $2\delta = 2(\lambda _k - \lambda _{k+1})$. Since $\delta > 0$, ${{\,\textrm{Hess}\,}}f$ will remain strictly positive definite in a neighbourhood of $\mathcal {V}_\alpha$ by continuity. To quantify this neighbourhood, we will connect $\mathcal {V}_\alpha$ to an arbitrary $\mathcal {X}$ using a geodesic and see how this influences the bounds of Lemma A.1. This also requires connecting $\mathcal {V}_\beta$ to $\mathcal {X}^\perp$. The next lemma shows that both geodesics are closely related. Recall that $\sin (t\theta )$ and $\cos (t\theta )$ denote diagonal matrices of size $k \times k$. For convenience, we will denote by *O* a zero matrix whose dimensions are clear from the context and is not always square.

### Lemma A.2

Let $X,Y \in \mathbb {R}^{n \times k}$ be such that $X^T X = Y^T Y = I_k$ with $k \le n/2$. Denote the principal angles between ${{\,\textrm{span}\,}}(X)$ and ${{\,\textrm{span}\,}}(Y)$ by $\theta _1 \le \cdots \le \theta _k$ and assume that $\theta _k< \pi /2$. Choose $X_\perp , Y_\perp \in \mathbb {R}^{n \times (n-k)}$ such that $X_\perp ^T X_\perp = Y_\perp ^T Y_\perp = I_{n-k}$ and ${{\,\textrm{span}\,}}(X_\perp ) = {{\,\textrm{span}\,}}(X)^\perp$, ${{\,\textrm{span}\,}}(Y_\perp ) = {{\,\textrm{span}\,}}(Y)^\perp$. Define the curves

$$\begin{aligned} \gamma (t)&:[0,1] \rightarrow \mathbb {R}^{n \times k} ,&t&\mapsto X V_1 \cos (t\theta ) + X_\perp V_2 \begin{bmatrix} O \\ \sin (t\theta ) \end{bmatrix}, \\ \gamma _\perp (t)&:[0,1] \rightarrow \mathbb {R}^{n \times (n-k)} ,&t&\mapsto X_\perp V_2 \begin{bmatrix} I \\   &  \cos (t\theta ) \end{bmatrix} - X V_1 \begin{bmatrix} O&\sin (t\theta ) \end{bmatrix}, \end{aligned}$$

where the orthogonal matrices $V_1,V_2$ are the same as in Lemma 4.3. Then ${{\,\textrm{span}\,}}(\gamma (t))$ is the connecting geodesic on ${{\,\textrm{Gr}\,}}(n,k)$ from ${{\,\textrm{span}\,}}(X)$ to ${{\,\textrm{span}\,}}(Y)$. Likewise, ${{\,\textrm{span}\,}}(\gamma _\perp (t))$ is a connecting geodesic on ${{\,\textrm{Gr}\,}}(n,n-k)$ from ${{\,\textrm{span}\,}}(X_\perp )$ to ${{\,\textrm{span}\,}}(Y_\perp )$. Furthermore, $\gamma (t)$ and $\gamma _\perp (t)$ are orthonormal matrices for all *t*.

### Proof

Assume $\theta _1 = \cdots = \theta _r = 0$, where $r=0$ means that $\theta _1 > 0$. Like in the proof of Prop. 3, the CS decomposition of *X* and *Y* from Lemma 4.3 can be written in terms of their principal angles $\theta _1, \ldots , \theta _k$. Since $\theta _k < \pi /2$ and $n \le k/2$, this gives after dividing certain block matrices the relations

$$\begin{aligned} Y^T X&= U_1 \, \cos (\theta ) \, V_1^T,&Y^T X_\perp&= U_1 \begin{bmatrix}O_{k \times (n-2k)}&\sin (\theta ) \end{bmatrix} V_2^T \\ Y_\perp ^T X&= U_2 \begin{bmatrix}O_{(n-2k) \times k} \\ \sin (\theta ) \end{bmatrix} V_1^T,&Y_\perp ^T X_\perp&= U_2 \begin{bmatrix}-I_{n-2k} \\   &  -\cos (\theta ) \end{bmatrix} V_2^T, \end{aligned}$$

where $U_1, V_1$ and $U_2,V_2$ are orthogonal matrices of size $k \times k$ and $(n-k) \times (n-k)$, resp.

Denote $\mathcal {X}={{\,\textrm{span}\,}}(X)$ and $\mathcal {Y}={{\,\textrm{span}\,}}(Y)$. By definition, the connecting geodesic $\gamma (t)$ is determined by the tangent vector ${{\,\textrm{Log}\,}}_{\mathcal {X}}(\mathcal {Y})$, which can be computed from (6). To this end, we first need the compact SVD of $M:= X_\perp X_\perp ^T Y (X^T Y)^{-1}$. Substituting the results from above, we get (cfr. (15))

$$\begin{aligned} M = X_\perp V_2 \begin{bmatrix} O_{(n-2k) \times k} \\ \sin (\theta ) \end{bmatrix} U_1^ T U_1 \, (\cos (\theta ))^{-1}\, V_1^T = X_\perp V_2 \begin{bmatrix} O_{(n-2k) \times k} \\ I_k \end{bmatrix} \, \tan (\theta ) \, V_1^T. \end{aligned}$$

Observe that this is a compact SVD. Applying (6), we therefore get

$$\begin{aligned} G:= {{\,\textrm{Log}\,}}_{\mathcal {X}}(\mathcal {Y}) = U \Sigma V^T \quad \text {with}\,U = X_\perp V_2 \begin{bmatrix} O \\ I_k \end{bmatrix}, \ \Sigma = \theta , \ V = V_1 \end{aligned}$$

and from (4), the connecting geodesic satisfies

$$\begin{aligned} {{\,\textrm{Exp}\,}}_{\mathcal {X}}(tG) = {{\,\textrm{span}\,}}( \, X V_1 \cos (t\theta ) + X_\perp V_2 \begin{bmatrix} O \\ I_k \end{bmatrix} \sin (t\theta ) \, ). \end{aligned}$$

We have proven the stated formula for $\gamma (t)$. Verifying that $\gamma (t)^T \gamma (t) = I_k$ follows from a simple calculation that uses $\cos ^2(t \theta ) + \sin ^2(t \theta ) = I_k$.

Denote $\mathcal {X}^\perp ={{\,\textrm{span}\,}}(X_\perp )$ and $\mathcal {Y}^\perp ={{\,\textrm{span}\,}}(Y_\perp )$. To prove $\gamma _\perp (t)$, we proceed similarly by computing $G^\perp := {{\,\textrm{Log}\,}}_{\mathcal {X}^\perp }(\mathcal {Y}^\perp )$, which requires now the SVD of $M^\perp := X X^T Y_\perp (X_\perp ^T Y_\perp )^{-1}$. Again substituting the results from the CS decomposition, we get

$$\begin{aligned} M^\perp&= X V_1 \begin{bmatrix}O_{k \times (n-2k)}&\sin (\theta ) \end{bmatrix} U_2^ T U_2 \begin{bmatrix} -I_{n-2k} \\   &  - \cos (\theta ) \end{bmatrix}^{-1} V_2^T \\&= X V_1 \begin{bmatrix}O_{k \times (n-2k)}&-\tan (\theta ) \end{bmatrix} V_2^T \end{aligned}$$

Since (6) requires a compact SVD with a *square* $\Sigma$, we rewrite this as

$$\begin{aligned} M^\perp = \begin{bmatrix} \widetilde{X}&X V_1 \end{bmatrix} \begin{bmatrix}O_{(n-2k) \times (n-2k)} \\   &  -\tan (\theta ) \end{bmatrix} V_2^T \end{aligned}$$

where $\widetilde{X}$ contains $n-2k$ columns that are orthonormal to *X* (the final result will not depend on $\widetilde{X}$). Let $\theta ^\perp _1 \le \cdots \le \theta ^\perp _{n-k}$ denote the principal angles between $\mathcal {X}^\perp$ and $\mathcal {Y}^\perp$. Up to zero angles, they are the same as those between $\mathcal {X}$ and $\mathcal {Y}$. Since $k \le n/2$, we thus have

$$\begin{aligned} \theta ^\perp _1 = \cdots = \theta ^\perp _{n-2k} = 0, \ \theta ^\perp _{n-2k+1} = \theta _1, \ldots , \theta ^\perp _{n-k} = \theta _k. \end{aligned}$$

Applying (6) with these principal angles, we obtain

$$\begin{aligned} G^\perp := {{\,\textrm{Log}\,}}_{\mathcal {X}^\perp }(\mathcal {Y^\perp }) = U \Sigma V^T \quad \text {with}\,U = -\begin{bmatrix} \widetilde{X}&X V_1 \end{bmatrix}, \ \Sigma = \theta ^\perp , \ V = V_2. \end{aligned}$$

From (4), the corresponding geodesic satisfies

$$\begin{aligned} {{\,\textrm{Exp}\,}}_{\mathcal {X}^\perp }(tG^\perp )&= {{\,\textrm{span}\,}}( \, X_\perp V_2 \cos (t\theta ^\perp ) - \begin{bmatrix} \widetilde{X}&X V_1 \end{bmatrix} \sin (t\theta ^\perp ) \, ) \\&= {{\,\textrm{span}\,}}( \, X_\perp V_2 \begin{bmatrix} I_{n-2k} \\   &  \cos (t\theta ) \end{bmatrix} - \begin{bmatrix} O_{n \times (n-2k)}&X V_1 \sin (t\theta ) \end{bmatrix} \, ). \end{aligned}$$

Rewriting the block matrix, we have proven $\gamma _\perp (t)$. Its orthonormality is again a straightforward verification. $\square$

With the previous lemma, we can now investigate the Riemannian Hessian of *f* near $\mathcal {V}_\alpha$ when it is given in the matrix form $H_X$ of Lemma A.1. Let $\mathcal {X} = {{\,\textrm{span}\,}}(X) \in {{\,\textrm{Gr}\,}}(n,k)$ with orthonormal *X*. Its principal angles with $\mathcal {V}_\alpha$ are $\theta _1 \le \cdots \le \theta _k < \pi /2$. Use the substitutions $X \mapsto V_\alpha , Y \mapsto X$ and $X_\perp \mapsto V_\beta , Y_\perp \mapsto X_\perp$ in Lemma A.2 to define the geodesics $\gamma (t)$ and $\gamma _\perp (t)$ that connect $\mathcal {V}_\alpha$ to $\mathcal {X}$, and $\mathcal {V}_\beta$ to $\mathcal {X}^\perp$, resp. Denoting

$$\begin{aligned} C:= \cos (\theta ), \ S:= \sin (\theta ), \ \widetilde{C}:= \begin{bmatrix} I \\   &  C \end{bmatrix}, \ \widetilde{S}:= \begin{bmatrix} O \\ S \end{bmatrix}, \end{aligned}$$

we get the following expressions for the geodesics:

$$\begin{aligned} \gamma (t) = V_\alpha V_1 C + V_\beta V_2 \widetilde{S}, \quad \gamma _\perp (t) = V_\beta V_2 \widetilde{C} - V_\alpha V_1 \widetilde{S}^T. \end{aligned}$$

Recall that $H_X$ is defined using $X^T A X$ and $X_\perp ^T A X_\perp$. Since $\gamma (1) = XQ_1$ and $\gamma _\perp (1) = X^\perp Q_2$ for some orthogonal matrices $Q_1, Q_2$, we can write with $A = V_\alpha \Lambda _{\alpha } V_\alpha ^T + V_\beta \Lambda _{\beta } V_\beta ^T$ that

33 $$\begin{aligned} \begin{aligned} Q_1^T X^T A X Q_1&= \gamma (1)^T A \gamma (1) \\&= C \, (V_1^T \Lambda _{\alpha } V_1) \, C + \widetilde{S}^T \, (V_2^T \Lambda _{\beta } V_2) \, \widetilde{S} \\ Q_2^TX_\perp ^T A X_\perp Q_2&= \gamma _\perp (1)^T A \gamma _\perp (1) \\&= \widetilde{C} \, (V_2^T \Lambda _{\beta } V_2) \, \widetilde{C} + \widetilde{S} \, (V_1^T \Lambda _{\alpha } V_1) \, \widetilde{S}^T. \end{aligned} \end{aligned}$$

Here we used simplifications like $V_\beta ^T AV_\alpha = V_\beta ^T V_\alpha \Lambda _{\alpha } = 0$.

A simple bounding of the eigenvalues of the difference of these matrices results in the main result.

### Theorem A.1

Let $k \le n/2$. Define the neighbourhood

$$\begin{aligned} B_* = \left\{ \mathcal {X} \in {{\,\textrm{Gr}\,}}(n,k) :\sin ^2 (\theta _k(\mathcal {X}, \mathcal {V}_\alpha )) \le \frac{\delta }{\lambda _1 + \lambda _k} \right\} , \end{aligned}$$

then *f* is geodesically convex on $B_*$.

### Proof

Our aim is to show that $\lambda _{i,j}(H_X)$ remains positive given the bound on $\theta _k$. From Lemma A.1, we see that

34 $$\begin{aligned} \lambda _{\min }(H_X) \ge 0 \quad \iff \quad \lambda _{\min }(X^T A X) \ge \lambda _{\max }(X_\perp ^T A X_\perp ). \end{aligned}$$

Since $Q_1,Q_2$ are orthogonal in (33), it suffices to find a lower and upper bound of, resp.,

$$\begin{aligned} \lambda _{\min }(X^T A X)&= \lambda _{\min }(C \, (V_1^T \Lambda _{\alpha } V_1) \, C + \widetilde{S}^T \, (V_2^T \Lambda _{\beta } V_2) \, \widetilde{S}) \\ \lambda _{\max }(X_\perp ^T A X_\perp )&= \lambda _{\max }(\widetilde{C} \, (V_2^T \Lambda _{\beta } V_2) \, \widetilde{C} + \widetilde{S} \, (V_1^T \Lambda _{\alpha } V_1) \, \widetilde{S}^T). \end{aligned}$$

Standard eigenvalue inequalities for symmetric matrices (see, e.g., [18, Cor. 4.3.15]) give

$$\begin{aligned} \lambda _{\min }(X^T A X)&\ge \lambda _{\min }(C \, (V_1^T \Lambda _{\alpha } V_1) \, C) + \lambda _{\min }(\widetilde{S}^T \, (V_2^T \Lambda _{\beta } V_2) \, \widetilde{S}) \\ \lambda _{\max }(X_\perp ^T A X_\perp )&\le \lambda _{\max }(\widetilde{C} \, (V_2^T \Lambda _{\beta } V_2) \, \widetilde{C}) + \lambda _{\max }(\widetilde{S} \, (V_1^T \Lambda _{\alpha } V_1) \, \widetilde{S}^T). \end{aligned}$$

Recall that $\lambda _1 \ge \cdots \ge \lambda _n$ are the eigenvalues of *A*. Since $\widetilde{S}$ is a tall rectangular matrix, we apply the generalized version of Ostrowski’s theorem from [16, Thm. 3.2] to each term above^6^ and obtain

$$\begin{aligned} \lambda _{\min }(C \, (V_1^T \Lambda _{\alpha } V_1) \, C)&\ge \lambda _{\min }(C^2) \lambda _{\min }(\Lambda _{\alpha }) = \cos ^2(\theta _k) \lambda _k \\ \lambda _{\min }(\widetilde{S}^T \, (V_2^T \Lambda _{\beta } V_2) \, \widetilde{S})&\ge \lambda _{\min }(\tilde{S}^T \tilde{S}) \lambda _{\min }(\Lambda _{\beta }) = \sin ^2(\theta _1) \lambda _n, \end{aligned}$$

since the matrices $V_1,V_2$ are orthogonal and $\theta _1 \le \cdots \le \theta _k < \pi /2$. Adding this gives the lower bound

35 $$\begin{aligned} \lambda _{\min }(X^T A X) \ge \cos ^2(\theta _k) \lambda _k + \sin ^2(\theta _1) \lambda _n \ge \cos ^2(\theta _k) \lambda _k. \end{aligned}$$

Likewise, using the block structure of $\widetilde{S}$, we get

$$\begin{aligned} \lambda _{\max }(\widetilde{C} \, (V_2^T \Lambda _{\beta } V_2) \, \widetilde{C})&\le \lambda _{\max }(C^2) \lambda _{\max }(\Lambda _{\beta }) = \cos ^2(\theta _1) \lambda _{k+1} \\ \lambda _{\max }(\widetilde{S} \, (V_1^T \Lambda _{\alpha } V_1) \, \widetilde{S}^T)&= \lambda _{\max }(S \, (V_1^T \Lambda _{\alpha } V_1) \, S) \\&\le \lambda _{\max }(S^2) \lambda _{\max }(\Lambda _{\alpha }) = \sin ^2(\theta _k) \lambda _1 \end{aligned}$$

and thus

36 $$\begin{aligned} \lambda _{\max }(X_\perp ^T A X_\perp ) \le \cos ^2(\theta _1) \lambda _{k+1} + \sin ^2(\theta _k) \lambda _1 \le \lambda _{k+1} + \sin ^2(\theta _k) \lambda _1. \end{aligned}$$

The condition (34) is thus satisfied when

$$\begin{aligned} \cos ^2(\theta _k) \lambda _k = \lambda _k - \sin ^2(\theta _k) \lambda _k \ge \lambda _{k+1} + \sin ^2(\theta _k) \lambda _1, \end{aligned}$$

which reduces to the bound on $\theta _k$ in the statement of the theorem.

It remains to show that $B_*$ is an open totally geodesically convex set. Since $\lambda _1 \ge \lambda _k \ge \lambda _{k+1} \ge 0$, we get

$$\begin{aligned} \frac{\lambda _k - \lambda _{k+1}}{\lambda _1 + \lambda _{k}} \le \frac{\lambda _k }{2\lambda _{k}} = \frac{1}{2}. \end{aligned}$$

Hence, $B_* = N_*(\varphi )$ with $\varphi \le \pi /4$ since $\sin ^2(\pi /4) = 1/2$. $\square$

If $k=1$, the proof above can be simplified.

### Corollary A.1

Let $k=1$ and define the neighbourhood

$$\begin{aligned} B_* = \left\{ \mathcal {X} \in {{\,\textrm{Gr}\,}}(n,1) :\sin ^2 (\theta _1(\mathcal {X}, \mathcal {V}_\alpha )) \le \frac{\delta }{\delta + \lambda _1 - \lambda _n} \right\} . \end{aligned}$$

Then *f* is geodesically convex on $B_*$.

### Proof

Since $k=1$, there is no need to simplify the bounds (35) and (36) as was done above. This gives that *f* is convex as long as

$$\begin{aligned} \cos ^2(\theta _1) \lambda _1 + \sin ^2(\theta _1) \lambda _n \ge \cos ^2(\theta _1) \lambda _{2} + \sin ^2(\theta _1) \lambda _1. \end{aligned}$$

Rewriting leads directly to the stated condition on $\sin ^2(\theta _1)$. $\square$

Remark that optimizing *f* on ${{\,\textrm{Gr}\,}}(n,1)$ is equivalent to

37 $$\begin{aligned} \min _{x \in \mathbb {R}^n} - x^T A x \qquad \text {s.t.} \quad \Vert x\Vert = 1, \end{aligned}$$

which is the minimization of the Rayleigh quotient problem on the unit sphere $S^{n-1} = \{ x \in \mathbb {R}^n :x^T x = 1 \}$. Cor. A.1 can therefore also be phrased in terms of a geodesically convex region for this problem. Denoting a unit norm top eigenvector of *A* by $v_1$ and using that $\sin ^2 \theta _1 = 1 - \cos ^2 \theta _1$, we get that (37) is geodesically convex on

$$\begin{aligned} \hat{B}_* = \left\{ x \in S^{n-1} :(x^T v_1)^2 \ge 1 - \frac{\delta }{\delta + \lambda _1 - \lambda _n} \right\} . \end{aligned}$$

This result can now be directly compared to [19, Lemma 7] where the corresponding region is defined as $(x^T v_1)^2 \ge 1 - \frac{\delta }{\delta + \lambda _1}$. This is a stricter condition and our result is therefore a small improvement.

## Appendix B: Convergence of Steepest Descent with step $\frac{1}{\gamma }$

We now prove convergence of steepest descent with a more tractable choice of step-size compared to the analysis of the main paper. However, this requires a slightly better initialization at most $\frac{\pi }{2 \sqrt{2}}$ away from the minimizer.

### Maximum extent of the Iterates

We first prove that steepest descent with step-size at most $\frac{1}{\gamma }$ does not guarantee contraction on distances from step to step, but we can still bound the distance at step *t* with the initial distance up to a scalar:

#### Proposition 6

Consider steepest descent applied to *f* with step-size $\eta \le \frac{1}{\gamma }$. If the iterates $\mathcal {X}_t$ satisfy $\theta _k(\mathcal {X}_t,\mathcal {V}_{\alpha })<\frac{\pi }{2}$, then they also satisfy

$$\begin{aligned} \text {dist}^2(\mathcal {X}_t, \mathcal {V}_{\alpha }) \le 2 \text {dist}^2(\mathcal {X}_0, \mathcal {V}_{\alpha }). \end{aligned}$$

#### Proof

Consider the discrete Lyapunov function

$$\begin{aligned} \mathcal {E}(t)= \frac{1}{\gamma } (f(\mathcal {X}_t)-f^*)+\frac{1}{2} \text {dist}^2(\mathcal {X}_t, \mathcal {V}_{\alpha }). \end{aligned}$$

Then

$$\begin{aligned} \mathcal {E}(t+1)-\mathcal {E}(t) = \frac{1}{\gamma }(f(\mathcal {X}_{t+1})-f(\mathcal {X}_t))+\frac{1}{2} ( \text {dist}^2(\mathcal {X}_{t+1},\mathcal {V}_{\alpha })-\text {dist}^2(\mathcal {X}_t,\mathcal {V}_{\alpha }) ). \end{aligned}$$

By $\gamma$-smoothness of *f*, we have

$$\begin{aligned}  &   f(\mathcal {X}_{t+1})-f(\mathcal {X}_t) \le \langle \text {grad}f(\mathcal {X}_t),\text {Log}_{\mathcal {X}_t}(\mathcal {X}_{t+1}) \rangle +\frac{\gamma }{2} \text {dist}(\mathcal {X}_t,\mathcal {X}_{t+1})^2 \\    &   \quad = \left( -\eta +\frac{\gamma }{2} \eta ^2 \right) \Vert \text {grad}f(\mathcal {X}_t) \Vert ^2. \end{aligned}$$

We also know by Proposition 3 that

$$\begin{aligned} \langle \text {grad}f(\mathcal {X}), -{{\,\textrm{Log}\,}}_{\mathcal {X}}(\mathcal {V_{\alpha })} \rangle \ge 0, \end{aligned}$$

for any $\mathcal {X}$ with $\theta _k(\mathcal {X},\mathcal {V}_\alpha ) < \pi /2$. By the fact that the sectional curvatures of the Grassmann manifold are non-negative, we have

$$\begin{aligned} \text {dist}^2(\mathcal {X}_{t+1},\mathcal {V}_{\alpha })&\le \text {dist}^2(\mathcal {X}_t,\mathcal {V}_{\alpha }) + \text {dist}^2(\mathcal {X}_{t+1},\mathcal {X}_t) - 2 \langle {{\,\textrm{Log}\,}}_{\mathcal {X}_{t}}(\mathcal {X}_{t+1}), {{\,\textrm{Log}\,}}_{\mathcal {X}_t}(\mathcal {V_{\alpha })} \rangle \\  &= \text {dist}^2(\mathcal {X}_t,\mathcal {V}_{\alpha }) + \eta ^2 \Vert \text {grad}f(\mathcal {X}_t) \Vert ^2 + 2 \eta \langle \text {grad}f(\mathcal {X}_t) , {{\,\textrm{Log}\,}}_{\mathcal {X}_t}(\mathcal {V_{\alpha })} \rangle \\  &\le \text {dist}^2(\mathcal {X}_t,\mathcal {V}_{\alpha }) + \eta ^2 \Vert \text {grad}f(\mathcal {X}_t) \Vert ^2. \end{aligned}$$

Thus

$$\begin{aligned}&\mathcal {E}(t+1)-\mathcal {E}(t) \le \left( -\frac{\eta }{\gamma }+\frac{\eta ^2}{2} \right) \Vert \text {grad}f(\mathcal {X}_t) \Vert ^2+ \frac{\eta ^2}{2}\Vert \text {grad}f(\mathcal {X}_t) \Vert ^2 \\  &\quad \le \left( -\frac{\eta }{\gamma }+\eta ^2 \right) \Vert \text {grad}f(\mathcal {X}_t) \Vert ^2 \le 0, \end{aligned}$$

because $\eta \le \frac{1}{\gamma }$. Since $\mathcal {E}(t)$ does not increase, we have

$$\begin{aligned} \frac{1}{2} \text {dist}^2(\mathcal {X}_t,\mathcal {V}_{\alpha })&\le \mathcal {E}(t) \le \mathcal {E}(0) = \frac{1}{\gamma } (f(\mathcal {X}_0)-f^*)+\frac{1}{2} \text {dist}^2(\mathcal {X}_0, \mathcal {V}_{\alpha }) \\  &\le \frac{1}{2} \text {dist}^2(\mathcal {X}_0, \mathcal {V}_{\alpha }) + \frac{1}{2} \text {dist}^2(\mathcal {X}_0, \mathcal {V}_{\alpha })= \text {dist}^2(\mathcal {X}_0, \mathcal {V}_{\alpha }) \end{aligned}$$

and the desired result follows. $\square$

### Convergence Under Positive Eigengap

When $\delta >0$, we can use gradient dominance to prove convergence of steepest descent to the (unique) minimizer in terms of function values:

#### Proposition 7

Steepest descent with step-size $\eta = 1/\gamma$ initialized at $\mathcal {X}_0$ such that

$$\begin{aligned} \text {dist}(\mathcal {X}_0,\mathcal {V}_{\alpha }) \le \frac{\pi }{4} \end{aligned}$$

satisfies

$$\begin{aligned} f(\mathcal {X}_t)-f^* \le \left( 1-0.32 c_Q \frac{\delta }{\gamma } \right) ^t (f(\mathcal {X}_0)-f^*). \end{aligned}$$

#### Proof

By the previous result and an induction argument to guarantee that the biggest angle between $\mathcal {X}_t$ and $\mathcal {V}_{\alpha }$ stays strictly less than $\pi /2$, we can bound the quantities $a(\mathcal {X}_t)$ uniformly from below:

Since $\text {dist}(\mathcal {X}_t,\mathcal {V}_{\alpha }) \le \sqrt{2} \cdot \text {dist}(\mathcal {X}_0,\mathcal {V}_{\alpha }) \le \frac{\sqrt{2} \pi }{4}$, we have

$$\begin{aligned} a(\mathcal {X}_t) \ge \cos (\theta _k(\mathcal {X}_t,\mathcal {V}_{\alpha })) \ge \cos (\text {dist}(\mathcal {X}_t,\mathcal {V}_{\alpha })) \ge \cos \left( \frac{\sqrt{2} \pi }{4} \right) \ge 0.4. \end{aligned}$$

By $\gamma$-smoothness of *f*, we have

$$\begin{aligned} f(\mathcal {X}_{t+1})-f(\mathcal {X}_t) \le -\frac{\Vert \text {grad}f(\mathcal {X}_t) \Vert ^2}{2 \gamma } \end{aligned}$$

and applying gradient dominance (Proposition 4), we get the bound

$$\begin{aligned} f(\mathcal {X}_{t+1})-f(\mathcal {X}_t) \le -\frac{2 c_Q \delta a^2(\mathcal {X}_t)}{\gamma }(f(\mathcal {X}_t)-f^*) \end{aligned}$$

thus

$$\begin{aligned} f(\mathcal {X}_{t+1})-f^* \le \left( 1-2 c_Q a^2(\mathcal {X}_t ) \frac{\delta }{\gamma }\right) (f(\mathcal {X}_t)-f^*) \le \left( 1-0.32 c_Q \frac{\delta }{\gamma } \right) (f(\mathcal {X}_t)-f^*). \end{aligned}$$

By induction the desired result follows. $\square$

We now state the iteration complexity of steepest descent algorithm:

#### Theorem B.1

Steepest descent with step-size $\frac{1}{\gamma }$ starting from a subspace $\mathcal {X}_0$ with distance at most $\frac{\pi }{4}$ from $\mathcal {V}_{\alpha }$ computes an estimate $\mathcal {X}_T$ of $\mathcal {V}_{\alpha }$ such that $\text {dist}(\mathcal {X}_T,\mathcal {V}_{\alpha })\le \epsilon$ in at most

$$\begin{aligned} T= \frac{1}{0.32 c_Q} \frac{\gamma }{\delta } \log \frac{f(\mathcal {X}_0)-f^*}{c_Q \delta \epsilon ^2}+1 \le {\mathcal {O}}\left( \frac{\gamma }{\delta } \log \frac{f(\mathcal {X}_0)-f^*}{\delta \epsilon } \right) . \end{aligned}$$

#### Proof

For $\text {dist}(\mathcal {X}_T,\mathcal {V}_{\alpha }) \le \epsilon$, it suffices to have

$$\begin{aligned} f(\mathcal {X}_T)-f^* \le c_Q \epsilon ^2 \delta \end{aligned}$$

by quadratic growth of *f* in Proposition 2. Using $(1-c)^T \le \exp (-c T)$ for all $T \ge 0$ and $0 \le c \le 1$, the previous result gives that it suffices to choose *T* as the smallest integer such that

$$\begin{aligned} f(\mathcal {X}_T)-f^* \le \exp \left( - 0.32 c_Q \frac{\delta }{\gamma } T \right) (f(\mathcal {X}_0)-f^*) \le c_Q \epsilon ^2 \delta . \end{aligned}$$

Solving for *T* and substituting $c_Q = 4/\pi ^2$, we get the required statement. $\square$

### Gap-Less Result

We also prove a convergence result for the function values when $\delta$ is assumed to be 0:

#### Theorem B.2

Steepest descent with step-size $\eta =\frac{1}{\gamma }$ initialized at $\mathcal {X}_0$ such that

$$\begin{aligned} \text {dist}(\mathcal {X}_0,\mathcal {V}_{\alpha }) \le \frac{\pi }{4} \end{aligned}$$

satisfies

$$\begin{aligned} f(\mathcal {X}_t)-f^* \le \frac{f(\mathcal {X}_0)-f^*+\frac{\gamma }{2}\text {dist}^2(\mathcal {X}_0, \mathcal {V}_{\alpha })}{0.4 t + 1} = {\mathcal {O}}\left( \frac{1}{t}\right) . \end{aligned}$$

#### Proof

By Proposition 6, we have that $\text {dist}(X_t,\mathcal {V}_{\alpha }) \le \frac{\sqrt{2} \pi }{4}$ and *f* satisfies the weak-quasi-convexity inequality at any iterate $\mathcal {X}_t$ of steepest descent with constant $C_0:=0.4$.

Consider the discrete Lyapunov function

$$\begin{aligned} \mathcal {E}(t)= \frac{C_0t+1}{\gamma } (f(\mathcal {X}_t)-f^*)+\frac{1}{2} \text {dist}^2(\mathcal {X}_t, \mathcal {V}_{\alpha }). \end{aligned}$$

We have that

$$\begin{aligned} \mathcal {E}(t+1)-\mathcal {E}(t) =&\frac{C_0t+C_0+1}{\gamma }(f(\mathcal {X}_{t+1})-f^*)-\frac{C_0t+1}{\gamma } (f(\mathcal {X}_t)-f^*) \\  &+\frac{1}{2}(\text {dist}^2(\mathcal {X}_{t+1},\mathcal {V}_{\alpha })-\text {dist}^2(\mathcal {X}_t,\mathcal {V}_{\alpha })). \end{aligned}$$

Now we have to estimate a bound for $\text {dist}^2(\mathcal {X}_{t+1},\mathcal {V}_{\alpha })-\text {dist}^2(\mathcal {X}_t,\mathcal {V}_{\alpha })$. By $\gamma$-smoothness of *f* and denoting $\Delta _t=f(\mathcal {X}_t)-f^*$ we have

$$\begin{aligned} \Delta _{t+1}-\Delta _t \le \langle \text {grad}f(\mathcal {X}_t),\text {Log}_{\mathcal {X}_t}(\mathcal {X}_{t+1}) \rangle +\frac{\gamma }{2} \text {dist}^2(\mathcal {X}_t,\mathcal {X}_{t+1})= -\frac{\Vert \text {grad}f(\mathcal {X}_t) \Vert ^2}{2\gamma } \end{aligned}$$

By $C_0$-weak–strong-convexity of *f* and the fact that the Grassmann manifold is of positive curvature, we have

$$\begin{aligned} C_0\Delta _t \le \frac{\gamma }{2}(\text {dist}^2(\mathcal {X}_t,\mathcal {V}_{\alpha })-\text {dist}^2(\mathcal {X}_{t+1},\mathcal {V}_{\alpha }))+\frac{ \Vert \text {grad}f(\mathcal {X}_t) \Vert ^2}{2\gamma } \end{aligned}$$

Summing this to the previous inequality, we get

$$\begin{aligned} \text {dist}^2(\mathcal {X}_{t+1},\mathcal {V}_{\alpha })-\text {dist}^2(\mathcal {X}_t,\mathcal {V}_{\alpha }) \le \frac{2}{\gamma } ((1-C_0)(f(\mathcal {X}_t)-f(\mathcal {X}_{t+1}))-C_0(f(\mathcal {X}_{t+1})-f^*)). \end{aligned}$$

Thus

$$\begin{aligned} \mathcal {E}(t+1)-\mathcal {E}(t)&\le \frac{C_0t+ 1 }{\gamma } (f(\mathcal {X}_{t+1})-f(\mathcal {X}_t))+\frac{C_0}{\gamma } (f(\mathcal {X}_{t+1})-f^*) \\  &+ \frac{1 - C_0}{\gamma } (f(\mathcal {X}_t)-f(\mathcal {X}_{t+1}))- \frac{C_0}{\gamma } (f(\mathcal {X}_{t+1})-f^*) \\  &=\frac{C_0t+C_0}{\gamma } (f(\mathcal {X}_{t+1})-f(\mathcal {X}_t)) \le 0. \end{aligned}$$

Thus $\mathcal {E}(t) \le \mathcal {E}(0)$ and the result follows. $\square$

## Footnotes

1 Krylov methods are arguably the most popular algorithms but they do not iterate on a subspace directly and are typically started from a single vector. In particular, they cannot easily improve a given approximation of a subspace for large $k>1$.

2 Personal communication by Yousef Saad.

3 This can be made very precise by describing ${{\,\textrm{Gr}\,}}(n,k)$ as the quotient of the Stiefel manifold with the orthogonal group. The elegant theory of this quotient manifold is worked out in [2].

4 Using the quotient manifold theory, one would use horizontal lifts.

5 The analysis of [19] is wrong with respect to this issue as discussed in detail in [4].
